# Modeling reaction–diffusion processes in simplified to complex astroglial branchlet geometries using different simulation tools

**DOI:** 10.1371/journal.pcbi.1014878

**Published:** 2026-10-28

**Authors:** Laura Keto, Saana Seppälä, Derek Ndubuaku, Annika Mäki, Tuomo Mäki-Marttunen, Marja-Leena Linne, Tiina Manninen

**Affiliations:** Faculty of Medicine and Health Technology, Tampere University, Tampere, Finland; East China University of Science and Technology, CHINA

## Abstract

Computational modeling of spatial biochemical processes in neuroscience is increasingly important as advances in experimental and imaging techniques provide detailed insights into cellular organization. Reaction–diffusion models are commonly used to study the diffusion and interactions of ions and molecules within neuronal cells, and multiple open-access simulation platforms have been developed to support such analysis. However, a comprehensive analysis of commonly used simulation tools, including MCell, STEPS, NeuroRD, and NEURON, remains lacking, particularly for realistic glial cell geometries, as these tools were originally developed for neurons. Here, we present a systematic analysis of these four simulation environments by implementing equivalent calcium diffusion and calcium–calmodulin reaction–diffusion models across a range of spatial domains. These include simplified geometries (cylinders and rectangular cuboids) as well as biologically realistic astroglial morphologies derived from electron microscopy data, including mouse Bergmann glia and mouse and human cortical astrocytes. Using matched kinetic parameters and diffusion constants, we examine simulation outputs and morphological variations across platforms under comparable conditions. We find that all tools produce overall consistent results for the systems considered, indicating that each can reliably capture diffusion and reaction–diffusion dynamics in simplified and complex geometries. At the same time, we show that the structural heterogeneity of branchlets from human astrocytes and mouse Bergmann glia gives rise to distinct calcium signaling, demonstrating that fine-scale morphology critically shapes astroglial dynamics and must be accurately captured by simulation frameworks. In addition, notable differences emerge in geometry representation, implementation strategies, computational aspects, and usability of the tools. By highlighting key trade-offs between geometric fidelity, flexibility, and computational cost in modeling intracellular biochemical processes, these findings provide practical guidance for selecting appropriate simulation tools. More broadly, our results support the use of reaction–diffusion modeling in morphologically realistic astrocytic environments and contribute to enabling mechanistic studies of localized signaling events at synaptic and perisynaptic sites.

## Author summary

Simulation of cellular reaction–diffusion systems is receiving considerable attention due to increasing computing power and biological knowledge. Several open-access simulation tools have been developed for this purpose and, in this study, we are interested in utilizing and examining four of them, MCell, STEPS, NeuroRD, and NEURON. With these four selected tools, we implement the same diffusion and reaction–diffusion models in cylinder, rectangular cuboid, and branchlets of real astroglial morphologies to perform the simulations. We conclude that the simulation results are consistent between the simulation tools. However, the tools differ in their usability, approaches to morphological representation and implementation, and the support for simulations in different dimensionalities. Understanding the properties and capabilities of these simulation tools will help develop better data-based models.

## Introduction

Advances in computational resources and experimental approaches to resolve spatiotemporal biochemical signaling at the subcellular level have attracted growing interest in reaction–diffusion modeling of brain systems, complementing and extending traditional reaction-based models. In particular, the complex morphologies of different glial cells, such as astrocytes [1–3], and their spatiotemporally organized signaling require more detailed models than single-compartmental models, which are currently the most common type of approach utilized in the field [4–8].

A range of open-access simulation tools have been developed for reaction–diffusion modeling. These tools can be divided into three categories: the tools based on Brownian dynamics, the tools based on the reaction–diffusion master equation [9], and the tools based on partial differential equations. In the first category of tools, the models are called particle-based microscopic models, where each molecule or ion is explicitly modeled in space. Each particle has an exact position in space and can move on the basis of Brownian motion. If a particle moves within the reaction radius of another particle, they can react with each other according to the defined reaction scheme. Such simulation tools include, for example, MCell (https://mcell.org) [10–13] and Smoldyn (https://www.smoldyn.org) [14]. In the second category of tools, the models are called voxel-based mesoscopic models based on reaction–diffusion master equation, where the volume is divided into smaller homogeneous well-mixed subvolumes, the number of each type of molecule and ion in each subvolume is stored (the exact location of each molecule or ion is not modeled), the reactions are modeled inside the subvolumes, and diffusion is modeled as a reaction between the subvolumes. These simulations extend the Gillespie algorithm [15,16], which numerically simulates the Markov process described analytically by the chemical master equation in a single subvolume [15], by diffusion between subvolumes. Such simulation tools include, for example, STEPS (https://steps.sourceforge.net) [17], NeuroRD (https://github.com/neurord) [18], and MesoRD (https://mesord.sourceforge.net) [19]. In the third category of tools, the models are presented with partial differential equation based compartmental modeling, where the diffusion is described as a flux between the adjacent compartments. Such simulation tools include, for example, NEURON (https://www.neuronsimulator.org/) [20], ASTRO (https://github.com/LeonidSavtchenko/Astro) [21], which is an extension of NEURON to model astrocytes, and GENESIS/Chemesis (http://genesis-sim.org/) [22,23]. There are also tools that combine these approaches, for example, the Virtual Cell tool (https://vcell.org) [24,25], which utilizes Smoldyn in stochastic simulations and partial differential equations in deterministic reaction–diffusion simulations, and MOOSE (https://moose.ncbs.res.in/) [26], which utilizes Smoldyn, the Gillespie algorithm, and partial differential equations. Previous studies have examined subsets of these tools, either by numerical benchmarking or qualitative analysis [17,18,27–29]. However, a comprehensive analysis of commonly used open-access tools across consistent reaction–diffusion models and realistic cellular morphologies remains lacking. In particular, no previous study has jointly applied MCell, STEPS, NeuroRD, and the NEURON rxd module [30] under comparable conditions in biologically realistic geometries. In addition to freely available and open-source simulation tools, commercial software platforms can be used for continuum reaction–diffusion modeling in complex geometries. In the present study, we focus on freely available tools that have been developed or widely applied for cellular and neuronal reaction–diffusion modeling.

Recent advances in volume electron microscopy (EM) have enabled the reconstruction of cellular morphologies at nanometer resolution, revealing the fine structural complexity of brain tissue. Large-scale datasets such as the MICrONS mouse cortex dataset [31], human cortical reconstructions [32], and mouse cerebellar datasets [33], among others, provide detailed geometries of neurons, astrocytes, synapses, and their spatial relationships, including the nanoscale organization of tripartite synapses. These datasets are increasingly accessible through platforms such as Neuroglancer (https://github.com/google/neuroglancer) and cloud-based repositories such as BossDB (https://bossdb.org), allowing the use of biologically realistic morphologies in computational modeling [34]. Various tools have been developed to facilitate visualization, transformation, and preprocessing of biological morphologies for simulations. These range from general-purpose three-dimensional (3D) modeling and mesh-processing environments such as Blender (https://www.blender.org/) and MeshLab [35] to simulation-oriented mesh-conditioning frameworks such as GAMer 2 [36]. In addition, several neuroscience-oriented tools support the analysis and reconstruction of cellular morphologies, including NeuroMorph [37], NeuroMorphoVis [38,39], and the Ultraliser framework to generate simulation-ready cellular meshes [40]. Alpha_Mesh_Swc additionally enables automated generation of watertight surface meshes from skeleton descriptions of neuronal and glial morphologies [41]. Specialized toolkits such as CellRemorph [42] provide additional functionality for transforming and manipulating astrocyte morphologies for computational simulations. Despite these advances, the integration of EM-derived geometries into reaction–diffusion simulations remains limited. However, most existing models rely on idealized geometries or focus on a single astrocytic microdomain [6], and the morphological diversity of astrocytes across brain regions and species has rarely been explored in spatial reaction–diffusion simulations. This is particularly notable given the pronounced structural differences observed between astrocyte populations, including cortical astrocytes and cerebellar Bergmann glia, as well as between rodent and human astrocytes.

MCell, STEPS, NeuroRD, and NEURON simulation tools have been utilized separately to study diffusion and reaction–diffusion processes in geometries of varying complexity, including realistic cellular morphologies. Particle-based simulators such as MCell have been applied to realistic neuronal substructures, including dendritic spines and synaptic microdomains [11,43,44]. These approaches have also been used to investigate astrocyte-associated diffusion microdomains, for example in simulations of cerebellar glomeruli where astrocytic sheathing processes shape extracellular diffusion, typically represented using simplified geometric abstractions [45,46]. More recently, Monte Carlo diffusion simulations have also been performed in 3D astrocyte morphologies reconstructed from confocal microscopy, demonstrating the influence of realistic cellular geometry on intracellular diffusion dynamics [47]. In parallel, tetrahedral mesh-based tools such as STEPS enable spatial reaction–diffusion simulations in complex 3D geometries [17]. Lately, studies have begun to incorporate aspects of astrocyte ultrastructure into spatial models, highlighting how the nanoscale morphology of fine astrocytic processes can influence the dynamics of intracellular calcium signaling [48,49]. In contrast, NeuroRD is generally limited to simplified geometries, and the NEURON rxd framework relies on tree-based morphologies that require substantial preprocessing of EM data, such as skeletonization and voxelization, to approximate complex structures. Savtchenko et al. [21] developed the ASTRO framework, in which astrocyte skeletons reconstructed from light microscopy images were populated with simplified nanoscale processes generated from statistical analysis of EM data, enabling biophysically detailed whole-cell simulation in NEURON. Computational models have also examined the calcium dynamics in cerebellar Bergmann glial branchlets, typically representing these processes as simplified cylindrical compartments in compartmental simulations [50]. As a result, the use of full EM-derived astrocyte morphologies—particularly fine branchlets with loop-like structures—remains technically challenging and relatively unexplored across simulation platforms. Studying astroglial structures with these tools therefore not only reveals differences in simulation approaches but also highlights current limitations in handling complex, non-tree-like cellular geometries. This is particularly relevant for astrocytes, whose nanoscale processes and spatial relationships with synapses play a central role in calcium signaling and biochemical compartmentalization. Thus, a systematic analysis of how different simulation tools handle such morphologies is needed to fully leverage the growing availability of high-resolution EM data in computational modeling.

Astrocytes extend fine processes that surround and partially ensheath synapses, forming the astrocytic component of the tripartite synapse and supporting highly localized signaling events. Ultrastructural analyses of astrocytic processes in the brain neuropil have shown that these branchlets are extremely thin structures with high surface-to-volume ratios, with reported surface-to-volume ratios for fine astrocytic processes of approximately 19–33 μm^-1^ [51]; similarly high surface-to-volume ratio microdomains have also been described in cerebellar Bergmann glial processes [50]. These perisynaptic astrocytic processes can detect synaptic activity through neurotransmitter-activated receptors, such as metabotropic glutamate receptors, and synaptically evoked signaling can trigger localized calcium elevations within astrocytic processes [52,53]. Nanoscale imaging studies further reveal pronounced local heterogeneity within astrocyte branchlets, including bulbous compartments connected by thinner shafts [54] and distinct structural motifs such as constrictions, expansions, and cores [3]. Such geometrical features can promote biochemical compartmentalization and influence the spread of intracellular calcium signals between neighboring compartments [48,54]. Although several EM datasets of human cortical tissue have recently become available, quantitative analysis of nanoscale astrocyte branchlet morphologies remains limited. Together, these observations suggest that local variations in branchlet caliber, volume, and connectivity may strongly shape intracellular diffusion and reaction dynamics, underscoring the importance of realistic branchlet geometries in models of astrocytic calcium signaling and downstream calcium-dependent biochemical processes.

In this study, we implement equivalent calcium diffusion and calcium–calmodulin reaction–diffusion models across multiple spatial domains, including simplified geometries (cylinders and rectangular cuboids) and realistic astroglial morphologies reconstructed from EM data. Simulations are performed across the four platforms wherever supported, enabling direct analysis of simulation outcomes and differences in geometry handling, dimensionality, and modeling workflows. In the one-dimensional (1D) case, we focus on NEURON and NeuroRD, while in 3D models, we primarily examine MCell, STEPS, and NEURON, with additional NeuroRD simulations in the rectangular cuboid geometry. We present the tools and the implemented morphologies and cellular mechanisms, followed by the simulated results and analysis of the applicability of the frameworks, as well as their implications for computational modeling of intracellular biochemical processes in complex astroglial geometries.

## Methods

This section represents the simulation tools, the preparation of geometries and cell morphologies for the simulations, as well as the chemical reactions, initial values, and parameters for the biochemical systems modeled, and the simulation setup.

### Simulation tools

In this study, we analyze four simulation tools available to model reaction–diffusion systems: MCell, STEPS, NeuroRD, and the NEURON rxd module. Basic information on these simulation tools is summarized in Table 1, and a general overview of the tools is presented in the following sections. In addition to simulator-specific visualization tools, all simulators provide text-based output or direct output through the supported user interfaces which were used in our study. For more detailed descriptions of the tools, see, for example, the relevant documentation (MCell: https://mcell.org, STEPS: https://steps.sourceforge.net, NeuroRD: https://github.com/neurord, and NEURON: https://www.neuronsimulator.org/).

**Table 1:** Details of the simulation tools. Several properties are listed for the MCell, STEPS, NeuroRD, and NEURON tools related to reaction–diffusion simulations. Abbreviations not explained in the text include STL for Stereolithography format and ASC for ASCII text format.

Tool	MCell^a^	STEPS^b^	NeuroRD^c^	NEURON^d^
**Version**	4	5.0.4	3.3.0	8.2.6
**Applicability**	Reaction–diffusion	Reaction–diffusion, electrical activity	Reaction–diffusion	Single cells, network of cells, reaction–diffusion
**Language**	C/C++	C/C++	Java	C/C++, Python
**User interface**	Python, GUI	Python	XML files	HOC, Python, GUI
**Morphology format**	Polygonal surface mesh (OBJ, STL)	Volumetric mesh (MSH)	Cubic voxel mesh	Skeletal (SWC, Neurolucida ASC)
**Det./Stoch.**	Stoch.	Det./Stoch.	Det./Stoch.	Det. (Stoch.)
**Reaction method**	Monte Carlo	Gillespie algorithm (Wmdirect, Tetexact), CVODE (TetODE), Runge–Kutta (Wmrk4)	Asynchronous tau-leaping with Gillespie algorithm	Implicit Euler, CVODE
**Diffusion method**	Brownian random walk	Gillespie algorithm (Tetexact), CVODE (TetODE)	Asynchronous tau-leaping with Blackwell stochastic algorithm	Finite volume discretization
**Availability of online manuals and tutorials**	Yes, but they reflect earlier work	Yes	Yes, especially for 1D/2D simulations	Yes, but they are still under development
**Visualization**	CellBlender	Visualization tool of STEPS	NeuroRDViz	NEURON GUI
**Parallelization** ^e^	No	Yes	No	Yes
**Number of models in ModelDB** ^f^	6	11	7	880

^a^https://mcell.org [10–13]; ^b^ https://steps.sourceforge.net [17]; ^c^ https://github.com/neurord [18,55]; ^d^ https://www.neuronsimulator.org/ [20,30]; ^e^ Here, parallelization means dividing a single simulation realization across multiple computational processes and does not include the concurrent execution of multiple realizations. All of the tools are able to execute independent stochastic realizations concurrently. ^f^ https://modeldb.science/ [56]

#### MCell.

MCell (Monte Carlo cell; https://mcell.org) [10–13] is a particle-based open-access stochastic simulation tool that can be used to perform diffusion and reaction–diffusion simulations in a 3D space. MCell is written in C/C++ and can be used by scripting with Python. MCell tracks each molecule and ion in the system individually and treats them as infinitely tiny points, and models their interactions with the Monte Carlo procedure, where reaction constants from the mass-action law are converted into reaction probabilities. The movements of molecules and ions are based on the Einstein diffusion equation, the solution of which is Green’s function and is simulated using the Brownian random walk.

In Brownian random walk, molecules and ions move with a random, jittery movement caused by constant collision with other particles. They can also be reflected or absorbed by surfaces, and react with other particles within the interaction radius. The time step length is the primary input parameter that defines the accuracy of the simulation. The Monte Carlo method draws a random number between 0 and 1, compares it with probability tables generated at the start of each simulation, and determines the events at each time step [11,12]. In contrast to previous versions of MCell, where models are designed with Model Definition Language (MDL), MCell3 and MCell4 include a CellBlender graphical user interface (GUI) on the 3D modeling software Blender (https://www.blender.org/), which facilitates the design and visualization of the morphologies and simulations. Since MCell4, the CellBlender interface has been favored over MDL. In MCell, designing a simulation can be divided into four parts: (1) morphology, (2) molecules and ions, and their release sites, (3) reactions, and (4) optimization. Projects run via CellBlender are saved as Blender (BLEND) files containing the geometry, chemical species, reaction and diffusion definitions, and simulation settings. With CellBlender, particle positions are saved at specified simulation intervals and can be replayed as an animation in Blender to visualize particle movement within the geometry.

#### STEPS.

STochastic Engine for Pathway Simulation (STEPS; https://steps.sourceforge.net) [17] is an open-access simulation tool that can be used to perform diffusion, reactions, and reaction–diffusion simulations. STEPS is written in C/C++, but the user can run the simulator with a Python interface. The most popular solvers in STEPS are the stochastic solvers Wmdirect for homogeneous stochastic systems without diffusion and Tetexact for stochastic reaction–diffusion simulations. STEPS also provides deterministic solvers, including the Runge–Kutta-based Wmrk4 solver for homogeneous systems and the CVODE-based TetODE solver for spatial reaction–diffusion simulations [17]. For stochastic reaction–diffusion systems, Tetexact extends the Gillespie stochastic simulation algorithm [15,16] to spatial systems by representing diffusion between adjacent tetrahedra as stochastic events. Thus, the morphology is divided into smaller subvolumes (tetrahedra). In each tetrahedron, the molecules and ions are equally distributed and can react with each other. They can diffuse to adjacent tetrahedra modeled as a reaction between two tetrahedra. The Gillespie algorithm uses propensity functions at each time step to randomly choose how long the next time step is and which reaction happens during that time step.

In STEPS, designing a simulation can be divided into four parts: (1) biochemical model with molecules, ions, reactions, and parameters, (2) morphology, (3) linking biochemical model with the morphology, and (4) random number generation. The biochemical model and simulation setup are defined in Python scripts, while the morphology is imported as a tetrahedral mesh. Tools such as CUBIT (https://cubit.sandia.gov) and Gmsh [57] can be used to generate tetrahedral meshes of the morphology and export them in formats supported by STEPS. The STEPS visualization toolkit supports plotting simulation results as time courses or spatial profiles, as well as visualizing species distributions within the 3D tetrahedral mesh during and after the simulation.

#### NeuroRD.

NeuroRD (https://github.com/neurord) [18] is an open-access simulation tool that can perform diffusion, reaction, and reaction–diffusion simulations. NeuroRD is written in Java, and the user can utilize a text-based Extensible Markup Language (XML) user interface since there is no GUI or supported scripting language available. In NeuroRD, each subvolume (segment) of the morphology is built using the x-, y-, and z-coordinates in 1D–3D, and simulations are run using an asynchronous tau-leaping approach that combines the exact Gillespie stochastic simulation algorithm and tau-leaping algorithm for reactions [58], and the Blackwell stochastic diffusion algorithm [55] within the asynchronous framework for diffusion. In the asynchronous tau-leaping framework, multiple reaction and diffusion events can occur at each time step. The diffusion constants can vary between subvolumes.

NeuroRD needs six files from the user for the morphology, reactions and parameter values for reactions and diffusion, initial conditions, stimulation protocol, output specifications, and a master file that specifies the name of the other files, as well as additional technical simulation details. The morphology file specifies the geometry by providing the shapes of the segments and their connections to cubic meshes. Visualization can be performed using a separate NeuroRDViz tool after the simulations are run.

#### NEURON.

NEURON (https://www.neuronsimulator.org/) [20] is an open-access simulation environment that can be used to model cellular mechanisms of single neurons and networks, including diffusion, reactions, and reaction–diffusion systems. The diffusion and reaction mechanisms can be modeled with a built-in NEURON rxd module that provides a simple way to model the mechanisms with coupled reaction–diffusion equations in 1D–3D [30]. The rxd module allows user-specified coefficients, for example, for reaction rates, and the possibility to use cylinders and skeletal morphologies of cells. NEURON is programmed with C/C++. The NEURON interpreter can be directly controlled through the High Order Calculator (HOC) language that has a C-like syntax or through a Python interface. Additional functionality, mechanisms, and other model components can be implemented using the NEURON Model Description Language (NMODL). In NEURON, each cell is modeled as a tree-like sequence of sections that are automatically or manually subdivided into segments for numerical accuracy. The simulator supports deterministic simulations and simulations where stochasticity is added via internal random number generators to the discrete-time events (such as neurotransmitter release) or, when using fixed time-step simulations, to the continuous-time variable states themselves (such as fractions of open versus closed channels in the segment). NEURON supports both fixed and adaptive time step simulations. Visualization can be performed with NEURON’s own GUI.

### Cell morphologies

Astrocyte branchlets are highly specialized cellular structures in which morphology plays a critical role in shaping local signaling dynamics and cellular function. As an example, Arizono et al. [54] demonstrated that astrocytic calcium signals are highly localized to nanoscale domains shaped by the fine morphology of perisynaptic processes. Consequently, realistic representations of cell geometries are essential for computational modeling of astrocytic processes. The simulation tools considered in this study require cell morphologies to be provided in different formats, as summarized in Table 1. We therefore used eight distinct morphologies, ranging from simple rectangular cuboid (shortly called as a cuboid) and cylindrical geometries to detailed, biologically realistic cellular structures (Fig 1 and Table 2). The first morphology is a cuboid with a width of 0.39824 μm, a height of 0.39824 μm, and a length of 9.956 μm (Fig 1A). The second morphology is a cylinder with a radius of 0.22468 μm and a length of 9.956 μm (Fig 1B). The cuboid and cylinder morphologies both have a volume of 1.579 μm^3^ and are defined in such a way that the cross-sectional areas of the morphologies are the same (Table 2). It is important to note that for 1D simulations, the shape of the cross-section does not affect the diffusion and reaction–diffusion results. Thus, we can reliably compare the results with a default geometry of a cuboid in NeuroRD and a default geometry of a cylinder in NEURON with identical volume and cross-sectional area. The cuboid and cylinder morphologies were built separately for NeuroRD and NEURON, but for MCell and STEPS in the same process explained in Section Mesh preparation for MCell and STEPS simulations.

**Table 2:** Morphometric properties of the simplified and astrocyte branchlet geometries used in the simulations. Real branchlet morphologies were obtained from previously published datasets [31–33]. Listed properties include total volume (V), surface area (SA), and surface-to-volume ratio (SVR). Abbreviations BG and CX refer to Bergmann glia and cortical astrocytes, respectively.

Tool	MCell and STEPS			NeuroRD			NEURON		
Property	V (μm^3^)	SA (μm^2^)	SVR (μm^-1^)	V (μm^3^)	SA (μm^2^)	SVR (μm^-1^)	V (μm^3^)	SA (μm^2^)	SVR (μm^-1^)
**Cuboid 1D**	–	–	–	1.579	16.177	10.245	–	–	–
**Cylinder 1D**	–	–	–	–	–	–	1.579	14.372	9.102
**Cuboid 3D**	1.568	15.676	10.000	1.577	16.177	10.258	–	–	–
**Cylinder 3D**	1.571	14.321	9.116	–	–	–	1.579	14.372	9.102
**Mouse BG 1**	0.657	10.017	15.254	–	–	–	0.691	10.840	15.680
**Mouse BG 2**	2.732	34.335	12.568	–	–	–	2.705	32.662	12.074
**Mouse CX 1**	0.714	14.625	20.496	–	–	–	0.838	16.006	19.107
**Mouse CX 2**	1.117	22.418	20.069	–	–	–	1.102	22.790	20.680
**Human CX 1**	1.189	13.519	11.375	–	–	–	1.218	14.168	11.633
**Human CX 2**	3.776	33.261	8.808	–	–	–	3.810	34.077	8.945

**Fig 1:**
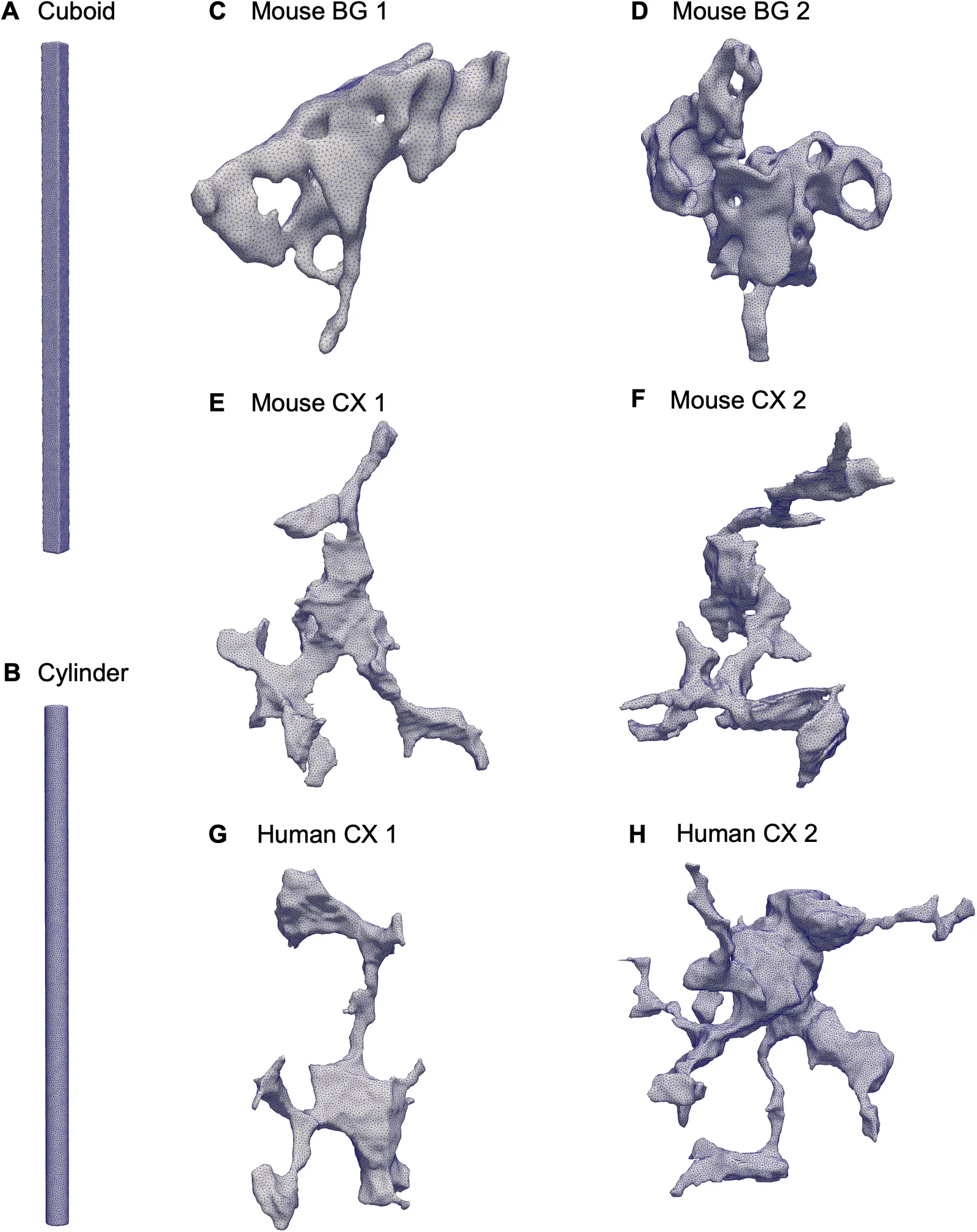
Simplified and astrocyte branchlet geometries used in the simulations. **(A)** Cuboid geometry with a width and height of 0.39824 μm and a length of 9.956 μm. **(B)** Cylinder geometry with a radius of 0.22468 μm and a length of 9.956 μm. **(C, D)** Mouse Bergmann glial branchlet morphologies 1 and 2 (Mouse BG 1 and 2) [33], with dimensions of 1.88 μm × 2.29 μm × 2.20 μm and 3.26 μm × 3.49 μm × 2.40 μm, respectively. **(E, F)** Mouse cortical astrocyte branchlet morphologies 1 and 2 (Mouse CX 1 and 2) [31], with dimensions of 3.59 μm × 2.20 μm × 4.70 μm and 4.06 μm × 3.49 μm × 4.39 μm, respectively. **(G, H)** Human cortical astrocyte branchlet morphologies 1 and 2 (Human CX 1 and 2) [32], with dimensions of 1.91 μm × 3.06 μm × 4.68 μm and 3.33 μm × 5.64 μm × 4.95 μm, respectively. Branchlet dimensions refer to the axis-aligned bounding-box extents (Δx×Δy×Δz) of the meshes shown. Additional morphometric properties are reported in Table 2. Morphologies are visualized with ParaView [59].

The six real cell morphologies are more complex and detailed 3D nanoprocess structures including two mouse Bergmann glial branchlet morphologies [33] (Fig 1C and 1D), two mouse cortical astrocyte branchlet morphologies [31] (Fig 1E and 1F), and two human cortical astrocyte branchlet morphologies [32] (Fig 1G and 1H). The morphometric properties of the geometries used in the simulations are shown in Table 2. The real geometries were reconstructed from EM data [31–33] and then imported into a suitable format for each simulation tool. We simulated all these branchlet morphologies in tools that support realistic 3D modeling: MCell, STEPS, and NEURON. Next, we explain in detail how the simplified geometries and branchlet morphologies were implemented in each tool.

#### Acquisition and processing of astrocyte morphologies.

The morphological data of the Bergmann glial branchlets were extracted from the serial EM dataset of the mouse cerebellar cortex (https://bossdb.org/project/nguyen_thomas2022) [33]. Bergmann glial segments were identified from the provided segmentation volume, and individual Bergmann glial process IDs were manually inspected using Neuroglancer (https://github.com/google/neuroglancer) to identify well-isolated lateral branches consistent with microdomain-scale processes. For each selected Bergmann glial process, a local 3D region of interest (ROI) was extracted from the segmentation volume using a custom Python pipeline based on CloudVolume [60]. ROI was defined as a cubic volume centered on the selected segment ID, with a half-extent of 5 μm in each spatial dimension and an additional edge padding of 8 voxels to avoid truncation artifacts. Only voxels belonging to a predefined whitelist of Bergmann glial segment IDs were retained. The segmentation mask within each ROI was converted into a smooth surface representation using a signed distance field (SDF)-based approach. First, a Euclidean distance transform was computed on the binary segmentation mask, followed by Gaussian smoothing with a standard deviation of 0.04 μm to suppress voxel-scale noise while preserving fine morphological features. To compensate for anisotropic EM voxel resolution, the volume was resampled anisotropically prior to surface extraction, using an upsampling factor of 2.5 in the x–y plane and 6.25 along the z-axis. Surface meshes were generated from the smoothed SDF using marching cubes and exported as triangular meshes.

Mouse cortical astrocyte branchlets were obtained from the MICrONS Consortium dataset (https://www.microns-explorer.org/), which provides automated segmentation of entire astroglial cells within a 1 mm^3^ of mouse visual cortex [31]. Using publicly released agglomerated segmentation data, complete astrocyte objects were identified by their unique segment IDs. The complete astrocyte meshes were downloaded and then imported into Blender to select two astrocyte branchlets with our CellRemorph toolkit [42].

Human cortical astrocyte branchlets were extracted from the petavoxel EM reconstruction of the human temporal cortex (https://h01-release.storage.googleapis.com/landing.html) [32]. This dataset encompasses a 1 mm^3^ volume of surgically excised human temporal cortex, imaged and segmented on the nanometer scale with automatic flood-filling network segmentation followed by agglomeration, including glial cell types such as astrocytes. Individual astrocyte segments were identified within this volume and their complete meshes were retrieved through Neuroglancer/CloudVolume. As with the mouse data, meshes were imported into Blender, where branchlets were selected with the CellRemorph toolkit [42].

#### Mesh preparation for MCell and STEPS simulations.

3D MCell simulations require closed polygonal surface meshes, whereas STEPS requires tetrahedral volumetric meshes. To support consistent representation of morphology, volume, and surface geometry across simulators, we adopted a unified mesh-preparation workflow. In summary, we first constructed surface meshes, transformed the surface meshes into volumetric meshes to be used in STEPS, and then converted the volumetric meshes back to surface meshes to be used in MCell. A more detailed description of the workflow is presented in the following.

The real astrocyte branchlet meshes obtained from the EM datasets were first converted into a standardized polygonal surface mesh representation in Object File Format (OFF). During this step, the meshes were cleaned and ensured to be watertight, two-manifold, and outward oriented while preserving the original morphology, surface area, and enclosed volume. The meshes were also triangulated where necessary so that all faces consisted of planar triangles suitable for downstream meshing tools.

For the simplified 3D cylinder and cuboid geometries, OFF surface meshes were generated procedurally using custom Python scripts. Cylindrical geometries were defined as right circular cylinders aligned with the z-axis, with radius r measured as 0.22468 μm and length L measured as 9.956 μm. The circular cross-section was discretized into 720 (n) evenly spaced circumferential samples, providing a high-fidelity polygonal approximation of the circular boundary. The lateral surface was tessellated into triangular strips and the top and bottom caps were closed using triangulated fans, producing watertight surfaces with consistent outward orientation. Cuboid geometries were defined by their widths wx, which is equal to 0.39824 μm, and wy, which is equal to 0.39824 μm, in the x- and y-directions and length L, which is equal to 9.956 μm, in the z-direction. The surface mesh was generated on a regular 3D lattice with a target surface edge length h as 0.07 μm, chosen to provide sufficiently fine surface discretization relative to the volumetric tetrahedral mesh used in the simulations while maintaining computational efficiency. Only the boundary nodes were retained and each quadrilateral surface patch was subdivided into two triangles, yielding a closed triangulated representation.

These OFF meshes were then converted into tetrahedral volume meshes using the CGAL Mesh_3-based MultiCompMesher tool (Okinawa Institute of Science and Technology Graduate University; https://github.com/CNS-OIST/MultiCompMesher). In our study, only a single interior component (cytosolic volume) was meshed. Tetrahedralization was performed using facet and cell sizing parameters selected to produce a uniform volumetric resolution, and CGAL’s built-in optimization procedures (ODT smoothing, Lloyd relaxation, and sliver perturbation/exudation) were applied to improve element quality. As a result, the total number of tetrahedra scaled with morphology size while maintaining a comparable discretization scale. The resulting tetrahedral meshes were exported to Gmsh ASCII Mesh (MSH) format and used directly for STEPS simulations. The final astroglial branchlet morphologies used in the STEPS simulations are shown in Fig 2.

**Fig 2:**
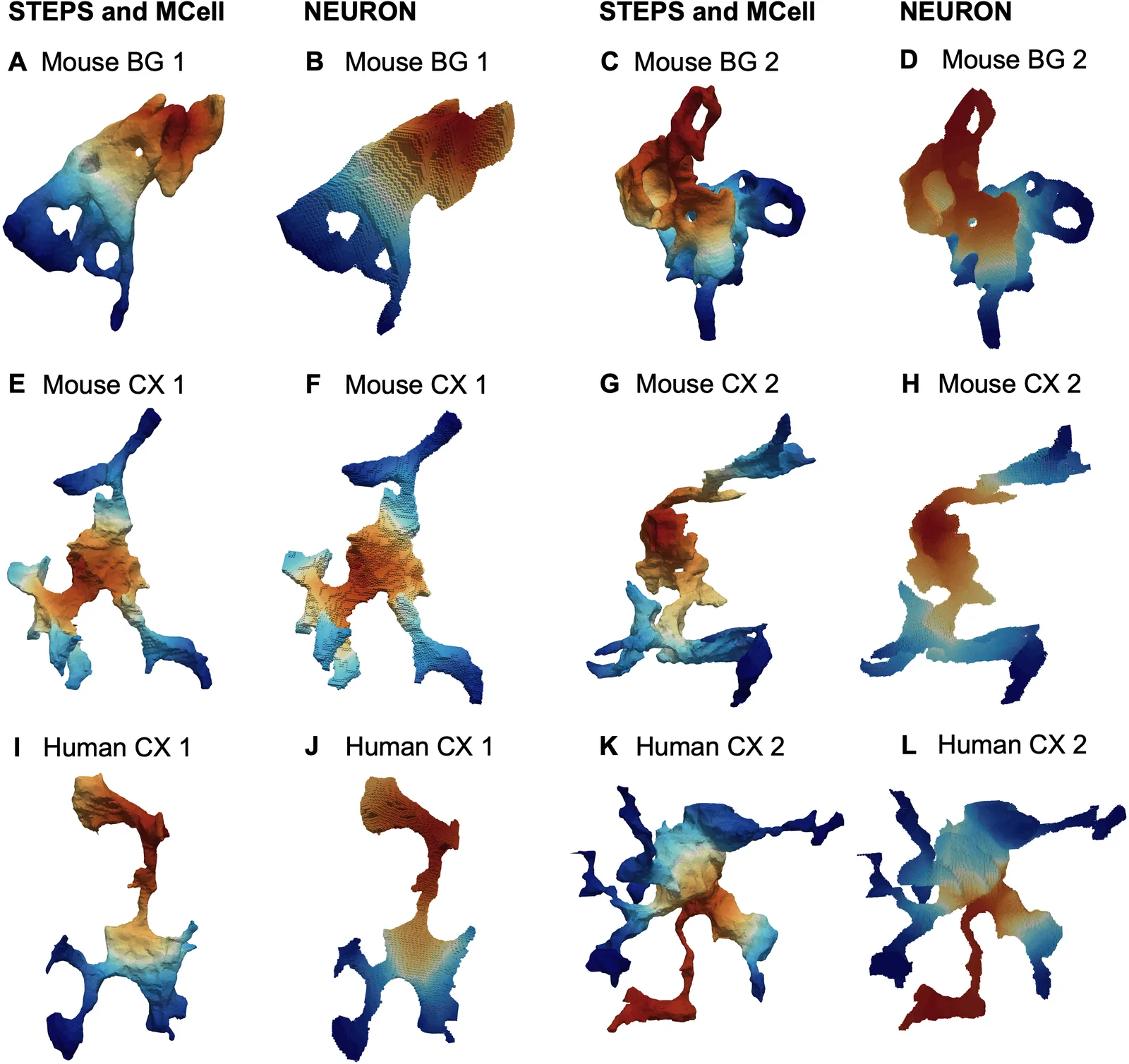
Astrocyte branchlet morphologies used in the 3D STEPS, MCell, and NEURON rxd simulations. **(A, B)** Mouse Bergmann glial branchlet morphology 1 (Mouse BG 1) [33]. **(C, D)** Mouse Bergmann glial branchlet morphology 2 (Mouse BG 2) [33]. **(E, F)** Mouse cortical astrocyte branchlet morphology 1 (Mouse CX 1) [31]. **(G, H)** Mouse cortical astrocyte branchlet morphology 2 (Mouse CX 2) [31]. **(I, J)** Human cortical astrocyte branchlet morphology 1 (Human CX 1) [32]. **(K, L)** Human cortical astrocyte branchlet morphology 2 (Human CX 2) [32]. Morphologies are shown after voxel-tree reconstruction and diameter scaling for NEURON simulations. The color indicates geodesic distance from the calcium injection site, discretized into 50 equal-volume bins, with red denoting the injection site and dark blue the most distal regions. Visualization was performed in ParaView [59].

To ensure geometric consistency between simulators, surface meshes in Wavefront Object (OBJ) format were extracted from the tetrahedral meshes used in STEPS. Where necessary, local surface defects were manually repaired and the meshes were triangulated before use in MCell. For each morphology, the enclosed volume and surface area of the final OBJ mesh were compared with those of the corresponding STEPS tetrahedral mesh and found to agree at the precision reported in Table 2. The number of subvolumes used in each geometry is provided in Table 3. In STEPS, the average size of the tetrahedral subvolume was chosen to provide sufficient spatial resolution to capture the complex morphology of the astrocyte branchlets while maintaining feasible computational cost. In all geometries, the mean tetrahedral volume ranged between 9.3 × 10^-6^ and 9.7 × 10^-6^ μm^3^, indicating a consistent volumetric discretization scale despite differences in the size and complexity of the morphologies. MCell simulations used the regenerated surface meshes directly and do not rely on an explicit volumetric subvolume discretization.

**Table 3:** Number of subvolumes in the simplified and astrocyte branchlet geometries. Values are for 3D case unless otherwise stated. Note that MCell does not utilize subvolumes during simulations, but for diffusion simulation plotting we utilized the same subvolume system as in STEPS for easy analysis. In 3D cuboid and cylinder simulations, the subvolume count in NeuroRD and NEURON is significantly lower compared to STEPS. Abbreviations used are BG for Bergmann glia and CX for cortex.

Morphology	MCell	STEPS	NeuroRD	NEURON rxd
**Cuboid**	1	164,309	1D: 25, 3D: 250	–
**Cylinder**	1	164,139	–	1D: 25, 3D: 30,250
**Mouse BG 1**	1	68,269	–	17,672
**Mouse BG 2**	1	287,373	–	60,468
**Mouse CX 1**	1	73,839	–	24,607
**Mouse CX 2**	1	116,798	–	31,845
**Human CX 1**	1	124,014	–	27,219
**Human CX 2**	1	406,199	–	80,846

#### Mesh preparation for NeuroRD simulations.

When coding geometric shapes in NeuroRD, the start and end x-, y-, and z-coordinates of each segment need to be specified in a separate morphology file. In practice, for a default cable-like cuboid geometry, only the x-coordinate differs between the segments. To create a straight geometry, the y- and z-coordinates are kept at the same level in the dimensionality, for example, at 0. Other specifications include a unique segment id, region type, radius (r), and optional label. To connect the adjacent segments and allow diffusion between them and the subvolumes, the starting x-coordinates of the next segment have to be initialized with the id of the previous segment, with “start on” and “end” specifications (e.g., start on=“seg1” at=“end”). In this study, we implemented with NeuroRD a cuboid morphology with 25 segments and, thus, with 25 subvolumes for the 1D models and with 50 segments and 250 subvolumes for the 3D models (Table 3). Each subvolume in the 1D case is the size of one determined segment to strictly confine the dimensionality to 1D. Specifically, each subvolume is a cubic of 0.39824 μm × 0.39824 μm × 0.39824 μm, determined with the x-coordinate and radius (0.19912 μm). Each segment and its connections are defined separately, possibly making the creation of more complex morphologies laborious unless automated tools are used. The number of subvolumes in 3D simulations (250) was determined based on the element size that allowed us to achieve the same total volume while preserving identical geometry dimensions used in NeuroRD 1D simulation as well as in the cuboid and cylinder simulations in other tools. In the 3D case, each segment was a cubic of 0.19912 μm × 0.39824 μm × 0.39824 μm, and each subvolume has dimensions of 0.19912 μm × 0.178 μm × 0.178 μm.

#### Morphology preparation for NEURON simulations.

When creating the cylinder morphology in NEURON, we have one section representing the whole geometry. The parameters given for the section are length (L = 9.956 μm), diameter (diam = 0.44936 μm), and number of segments (nseg), which defines the spatial discretization of the section into computational compartments. By default, each subvolume in the 1D simulation is the size of a segment, which is automatically divided by NEURON based on the length of the geometry and the number of nseg which we chose to be 25. Thus, in 1D simulations we had 25 segments and 25 rxd calcium nodes created using the Python interface for the NEURON h submodule (neuron.h) and the NEURON rxd module, representing the same cross-sectional area as was used in NeuroRD for the cuboid morphology. The diffusion occurs through these calcium nodes, which by default are the segment centers in the 1D case. When nodes are distributed in a 3D space, diffusion is independent of the number of segments, so we used nseg as 1. In this study, we call the segments and calcium nodes bins and subvolumes, respectively. To solve simulations with a 3D cylinder morphology, the geometry was defined as a list of 3D points using the built-in function pt3dadd, which specifies the x-, y-, and z-coordinates of the structure. NEURON’s 3D finite-volume solver (NEURON 3D rxd) was used to solve the reaction–diffusion system in 3D.

To ensure geometric consistency between STEPS and NEURON simulations with realistic astrocyte branchlet morphologies, the OBJ surface meshes were converted into voxel-tree Stockley–Wheal–Cannon (SWC) morphologies [61]. The meshes were voxelized using filled volumetric voxelization at a resolution of 0.04 μm with custom Python scripts based in part on the trimesh library (https://trimesh.org/). Minor discontinuities were corrected by a single morphological closing step (radius = 1 voxel). Voxel adjacency was defined using 6-connectivity for all morphologies except the human cortical astrocyte branchlet morphologies, for which 26-connectivity was required to preserve diagonal voxel contacts and prevent fragmentation of thin processes that would otherwise become disconnected under stricter connectivity constraints. The occupied voxel set was converted into a tree-structured skeleton, and local radii were assigned based on voxel occupancy with a minimum radius constraint of 0.03 μm. The resulting morphologies were exported in SWC format and imported into NEURON. Within NEURON, the morphologies were represented as constructive solid geometries composed of connected frusta, and intracellular diffusion was solved using NEURON 3D rxd on an internal Cartesian voxel grid. A uniform spatial discretization of dx = 0.04 μm was used for all morphologies. This resolution ensured consistent discretization across simulations while maintaining numerical stability and tractable simulation times, and allowed multiple spatial elements to span typical astrocyte branchlet diameters (approximately 0.1–0.3 μm). In NEURON 3D rxd, intracellular regions are discretized onto a Cartesian voxel grid with spacing dx, where each voxel corresponds to a computational node located at the center of the voxel. Reaction–diffusion dynamics are solved using a finite-volume formulation assuming uniform concentration within each voxel. Voxels fully contained within the intracellular region have a volume of dx^3^, whereas the voxels intersecting the morphology boundaries have fractional intracellular volumes estimated by subvoxel sampling of the constructive SWC geometry. Consequently, voxel volumes vary near boundaries according to the fraction of the voxel lying inside the intracellular domain. Because discretization of curved morphological boundaries on the Cartesian grid introduced morphology-dependent deviations in total volume, radii were uniformly scaled by morphology-specific factors to match the original mesh volumes as closely as possible. The diameter scaling factors were 0.40 and 0.70 for mouse Bergmann glial branchlet morphologies 1 and 2, respectively; 0.405 and 0.35 for mouse cortical astrocyte branchlet morphologies 1 and 2, respectively; and 0.45 for both human cortical astrocyte branchlet morphologies. The resulting morphometric properties, including volumes, surface areas, surface-to-volume ratios, and numbers of subvolumes, are reported in Tables 2 and 3. Because NEURON employs voxel-based discretization while STEPS uses tetrahedral meshes, the resulting number of spatial elements differs between the two simulators.

#### Geodesic computing of 3D calcium diffusion in realistic morphologies.

To enable consistent analysis of calcium diffusion across STEPS, MCell, and NEURON rxd, spatially resolved 3D calcium distributions were reduced to a 1D coordinate following the interior of each astrocyte morphology. In highly tortuous astrocyte branchlet geometries, diffusion distances must be evaluated along accessible intracellular pathways rather than straight-line Euclidean distances. Therefore, distances were computed as the internal shortest paths (geodesic distances) within each morphology. This geodesic coordinate system allowed diffusion profiles to be compared across simulators employing different representations of intracellular space—tetrahedral volume meshes in STEPS, particle-based diffusion within surface-defined volumes in MCell, and cubic finite-volume voxel grids in NEURON.

Fig 2 shows the color-coded geodesic distance from the calcium injection site for astrocyte branchlet morphologies used in STEPS, MCell, and NEURON diffusion simulations. For visualization of the NEURON morphologies in this figure, voxel volumes returned by NEURON were rendered as cuboid elements with volumes equal to the corresponding voxel volumes. Partial boundary voxels were displayed as scaled cuboids attached to neighboring voxels to reduce visual discontinuities while preserving the exact voxel volumes used in the NEURON simulations. This visualization procedure affects only the graphical representation and does not modify the numerical discretization or voxel volumes used in diffusion calculations.

For each discretization, a connectivity graph was constructed over volumetric elements (tetrahedra or voxels), with adjacency defined by shared faces. The element closest to the coordinate used for calcium injection in the simulations was designated as the source element for geodesic distance calculations. Geodesic distances from this source were computed using Dijkstra’s algorithm [62], to ensure that the distances reflect accessible intracellular diffusion pathways. Elements were then ordered by geodesic distance and partitioned into 50 iso-volume bins based on cumulative volume. Each bin was assigned a volume-weighted mean geodesic distance, and calcium ion counts were accumulated per bin at matched simulation time points. Geodesic-distance computation, bin construction, and cross-simulator profile analysis were implemented with custom Python scripts using NumPy [63] and SciPy [64].

In STEPS, molecule counts were obtained directly from tetrahedral observables. In MCell, particle coordinates were mapped onto the same tetrahedral mesh used for STEPS to ensure bin consistency. In NEURON rxd, voxel concentrations were converted to molecule counts using the corresponding voxel volumes before bin aggregation. This unified geodesic binning framework allowed diffusion profiles to be analyzed consistently across simulators despite their different spatial discretization schemes.

### Cellular mechanisms

We implemented the diffusion and reactions of the calcium–calmodulin pathway model, presented in this section, into all four simulation tools and simulated the calcium diffusion and the combined reaction–diffusion system in the different morphologies. All of the different molecules and ions were able to diffuse in the reaction–diffusion model. In MCell, all molecules and ions were defined as volume molecules in the CellBlender’s *Molecule* tab together with their diffusion constants. The injection location for calcium and the number of calcium ions and calmodulin molecules were determined under *Molecule Placement*, and the reactions were defined under *Reactions*. We left all the parameters concerning the interaction radius at their defaults. A reflective surface class was assigned to all interior surfaces of the geometries so that diffusing molecules remained confined within the morphology. In the other tools, the models were implemented according to the documentation and scripting language by utilizing the built-in mechanisms to define the molecules, diffusion constants, and reactions.

#### Diffusion model.

We examined the tools with 1D and 3D calcium diffusion by releasing 5,000 calcium ions at one end of the cylinder and cuboid morphologies (see Fig 1) and simulating diffusion for 1 ms. In these simplified geometries, the release site was defined along the central axis at coordinates (0, 0, 0.1) μm, corresponding to a point 0.1 μm inward from the tip of the structure. Using the same setup, we also examined three of the tools in realistic cell morphologies, where 5,000 calcium ions were released near a candidate synaptic contact site identified by inspection of the EM reconstruction of the astrocyte morphology. To approximate a localized calcium signal that occurs near synaptic activity while ensuring that the release volume remained fully inside the cell, the injection center was positioned approximately 0.1 μm from the putative synaptic contact site. The target injection volume was 0.001 μm^3^, corresponding to a sphere of diameter 0.124 μm.

In 3D STEPS and NEURON models, the injection region was defined using a matched volume-based procedure: starting from the source tetrahedron or voxel nearest to the chosen coordinate, neighboring spatial elements were added in order of center-to-center distance until the cumulative volume reached the target injection volume. In STEPS, the ions were distributed across the selected tetrahedra in proportion to the tetrahedron volume. In the 3D NEURON model, the selected voxel region was initialized to a uniform calcium concentration corresponding to the same total amount in the same target volume. In MCell, calcium was released within a sphere with a diameter of 0.124 μm centered at the same location. In NeuroRD, injection was defined as a concentration-based input over time. In 1D NEURON simulations, the injection site corresponded to a single spatial node. The diffusion constants used in the diffusion and reaction–diffusion simulations are listed in Table 4.

**Table 4:** Values for calcium (Ca^2+^) and calmodulin (CaM) diffusion parameters. The same value of *D*_CaM_ is used for calmodulin and all the calcium–calmodulin complexes.

Parameter	Value	Reference
*D*_Ca_	$2.2\times 10^{-10}$ $\frac{m^{2}}{s}$	[65]
*D*_CaM_	$1.1\times 10^{-11}$ $\frac{m^{2}}{s}$	[66]

#### Reaction–diffusion model.

We implemented the same calcium–calmodulin reaction pathway model described in the previous study [65] across all simulation tools (Table 5). Calmodulin contains four calcium-binding sites, two in the C-lobe and two in the N-lobe. The system was modeled as a closed reaction–diffusion system that included only diffusion and biochemical reactions, without membrane fluxes or endoplasmic reticulum mechanisms.

**Table 5:** Calcium–calmodulin signaling pathway model [65]. Calmodulin (CaM) has four binding sites for calcium (Ca^2+^), two in the C-lobe and two in the N-lobe.

Reaction	*k*_f_	*k*_b_
$Ca^{2+}+CaM\overset{k_{f}}{\underset{k_{b}}{⇌}}CaCaM_{N0C1}$	$6.8\times 10^{6}$ $\frac{1}{Ms}$	68 $\frac{1}{s}$
$Ca^{2+}+CaCaM_{N0C1}\overset{k_{f}}{\underset{k_{b}}{⇌}}Ca_{2}CaM_{N0C2}$	$6.8\times 10^{6}$ $\frac{1}{Ms}$	10 $\frac{1}{s}$
$Ca^{2+}+CaCaM_{N1C0}\overset{k_{f}}{\underset{k_{b}}{⇌}}Ca_{2}CaM_{N1C1}$	$6.8\times 10^{6}$ $\frac{1}{Ms}$	68 $\frac{1}{s}$
$Ca^{2+}+Ca_{2}CaM_{N1C1}\overset{k_{f}}{\underset{k_{b}}{⇌}}Ca_{3}CaM_{N1C2}$	$6.8\times 10^{6}$ $\frac{1}{Ms}$	10 $\frac{1}{s}$
$Ca^{2+}+Ca_{2}CaM_{N2C0}\overset{k_{f}}{\underset{k_{b}}{⇌}}Ca_{3}CaM_{N2C1}$	$6.8\times 10^{6}$ $\frac{1}{Ms}$	68 $\frac{1}{s}$
$Ca^{2+}+Ca_{3}CaM_{N2C1}\overset{k_{f}}{\underset{k_{b}}{⇌}}Ca_{4}CaM_{N2C2}$	$6.8\times 10^{6}$ $\frac{1}{Ms}$	10 $\frac{1}{s}$
$Ca^{2+}+CaM\overset{k_{f}}{\underset{k_{b}}{⇌}}CaCaM_{N1C0}$	$1.08\times 10^{8}$ $\frac{1}{Ms}$	4150 $\frac{1}{s}$
$Ca^{2+}+CaCaM_{N1C0}\overset{k_{f}}{\underset{k_{b}}{⇌}}Ca_{2}CaM_{N2C0}$	$1.08\times 10^{8}$ $\frac{1}{Ms}$	800 $\frac{1}{s}$
$Ca^{2+}+CaCaM_{N0C1}\overset{k_{f}}{\underset{k_{b}}{⇌}}Ca_{2}CaM_{N1C1}$	$1.08\times 10^{8}$ $\frac{1}{Ms}$	4150 $\frac{1}{s}$
$Ca^{2+}+Ca_{2}CaM_{N1C1}\overset{k_{f}}{\underset{k_{b}}{⇌}}Ca_{3}CaM_{N2C1}$	$1.08\times 10^{8}$ $\frac{1}{Ms}$	800 $\frac{1}{s}$
$Ca^{2+}+Ca_{2}CaM_{N0C2}\overset{k_{f}}{\underset{k_{b}}{⇌}}Ca_{3}CaM_{N1C2}$	$1.08\times 10^{8}$ $\frac{1}{Ms}$	4150 $\frac{1}{s}$
$Ca^{2+}+Ca_{3}CaM_{N1C2}\overset{k_{f}}{\underset{k_{b}}{⇌}}Ca_{4}CaM_{N2C2}$	$1.08\times 10^{8}$ $\frac{1}{Ms}$	800 $\frac{1}{s}$

We simulated the calcium–calmodulin reaction–diffusion system in the eight morphologies for 1 s with 4,000 calmodulin molecules initially distributed uniformly throughout the morphology. Calcium release was identical to that used in the diffusion-only simulations described above. After initialization, all molecules were free to diffuse and react according to the specified reaction scheme. The diffusion constant for calmodulin was not provided in [65], so we used the value reported in [66] (Table 4) for all calmodulin species regardless of the number of calcium ions bound.

### Simulation setup

All simulations were performed locally on a Mac operating system with an Apple M4 Pro chip and 24 GB of RAM. Each reaction–diffusion model is simulated for 1 s, and diffusion models for 1 ms. In diffusion simulations, the calcium count is plotted as a function of distance from the injection site with five time points: 0.05 ms, 0.1 ms, 0.2 ms, 0.5 ms, and 1 ms. In 1D diffusion simulations, distances were computed using axial distance along the cable. For 3D diffusion simulations in cuboid and cylinder geometries, the distances were defined using axial binning along the length of the geometry. In diffusion simulations with astrocyte branchlet morphologies, distances were computed using geodesic distance within the intracellular domain. For plotting the results from the 1D and 3D diffusion simulations, we divided the geometries into 25 and 50 bins, respectively, and plotted each data point in the middle of the bin width. In NEURON and MCell, we used 100,001 time points of length 10^-5^ s in the reaction–diffusion simulations, and used the same amount of points and time step when saving data from all the tools. The number of subvolumes used in STEPS, NeuroRD, and NEURON simulations is presented in Table 3. For stochastic simulations with MCell and STEPS, we also made 20 runs to calculate the mean and standard deviation with the astrocyte branchlet morphologies. The mean of stochastic simulations from MCell and STEPS or deterministic simulations from NEURON and STEPS with the astrocyte branchlet morphologies are compared with the root mean square deviation (RMSD), normalized root mean square deviation (NRMSD), and Pearson Correlation Coefficient methods. All the data points were used when calculating these values. The RMSD method is represented with the equation


$$\begin{array}{c} \text{RMSD}=\sqrt{\frac{1}{n}\sum_{i=1}^{n}|x(i)-y(i){|}^{2}}, \end{array}$$ (1)

where *x* and *y* are the *n* data points of the ion or molecule count from the deterministic simulation or from the mean of 20 stochastic simulations. *n* is 50 for diffusion simulations and 100,001 for reaction–diffusion simulations.

The NRMSD is calculated by dividing the RMSD by range of MCell or STEPS simulation values


$$\begin{array}{c} \text{NRMSD}=\frac{\text{RMSD}}{\max_{i}y(i)-\min_{i}y(i)}\times 100\%, \end{array}$$ (2)

where *y* is the same as in the previous equation, *n* data points of the ion or molecule count from the deterministic simulation or from the mean of 20 stochastic simulations. When comparing MCell to other tools, we use *y* from MCell simulations, and otherwise *y* is from STEPS simulations (either stochastic or deterministic).

To test the similarity in the pattern of two simulation tool results, we used Pearson Correlation Coefficient


$$\begin{array}{c} \text{Pearson}=\frac{\sum_{i=1}^{n}(x(i)-\bar{x})(y(i)-\bar{y})}{\sqrt{\sum_{i=1}^{n}(x(i)-\bar{x}{)}^{2}}\sqrt{\sum_{i=1}^{n}(y(i)-\bar{y}{)}^{2}}}, \end{array}$$ (3)

where *x* and *y* are as before the *n* data points of the ion or molecule count from the deterministic simulation or from the mean of 20 stochastic simulations, and $\bar{x}$ and $\bar{y}$ are single mean values of those. In this case, we also used *n* as 50 for diffusion simulations and 100,001 for reaction–diffusion simulations.

## Results

We first present simulation results across different morphologies, followed by an evaluation of the usability and suitability of the tools for modeling the associated dynamical behavior of the signaling pathway in the morphologies.

### Calcium dynamics in simplified and complex astrocyte geometries

#### Calcium diffusion simulations.

In this section, the results are presented as the total number of calcium ions as a function of the distance from the calcium injection site, that is, from one end of the geometry with cuboid and cylinder morphologies and from one possible synapse location with the realistic cellular morphologies, with five time points: 0.05 ms, 0.1 ms, 0.2 ms, 0.5 ms, and 1 ms.

**1D calcium diffusion in simplified geometries.** Fig 3 shows that 1D calcium diffusion was highly similar in NeuroRD and NEURON simulations. Both simulators employed 25 subvolumes (bins); see Table 2 and Fig 1A and 1B for morphologies. This was expected for a 1D simulation with large subvolumes in geometries with equivalent cross-sectional area, although diffusion in NEURON is a deterministic process, and in NeuroRD it is stochastic.

**Fig 3:**
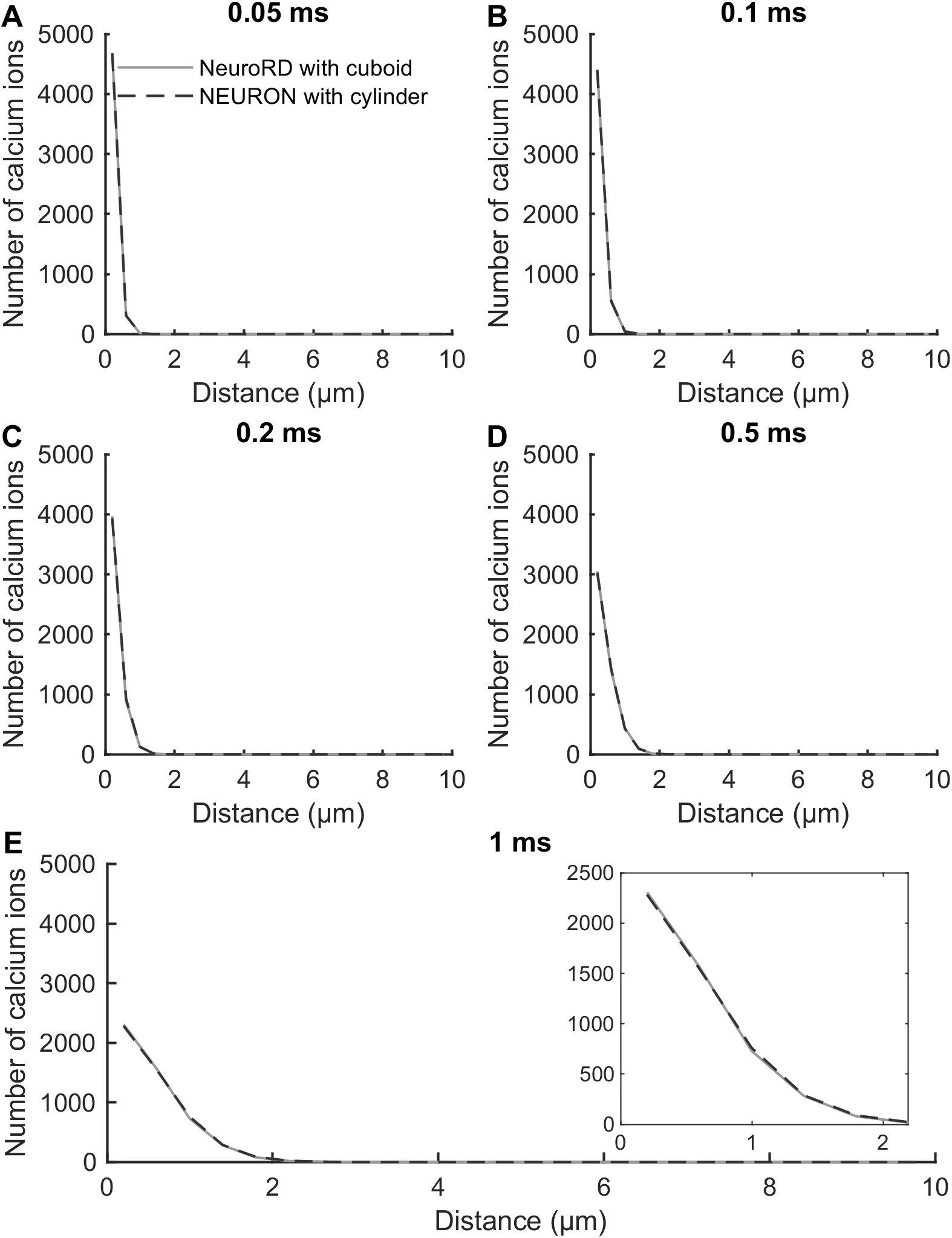
1D calcium diffusion results are consistent across NeuroRD and NEURON rxd simulations in the simplified geometries with 25 subvolumes. NeuroRD simulation shows a single realization of a stochastic run together with deterministic NEURON simulation. Number of calcium ions plotted as a function of distance from the injection point at time points of **(A)** 0.05 ms, **(B)** 0.1 ms, **(C)** 0.2 ms, **(D)** 0.5 ms, and **(E)** 1 ms with an inset.

**3D calcium diffusion in simplified geometries.** Fig 4 presents 3D calcium diffusion results with 50 distance bins created with all four tools, MCell, STEPS, NeuroRD, and NEURON, using cuboid and cylinder morphologies shown in Fig 1A and 1B. For stochastic simulations, we show only a single realization. Generally, the results from cuboid and cylinder morphologies in the same tool are consistent toward the end of the simulation, indicating that, despite slight differences in the shape, the behavior of the diffusion is similar. However, the results reveal some tool-to-tool variation. The simulation results from different tools with the cylinder morphology are often more similar than those with the cuboid morphology. Of these simulations, NeuroRD has the least number of subvolumes which may contribute to faster diffusion compared to the other tools (Table 3). Deterministic STEPS simulations show different results compared to the single realizations of stochastic STEPS simulations and other tools.

**Fig 4:**
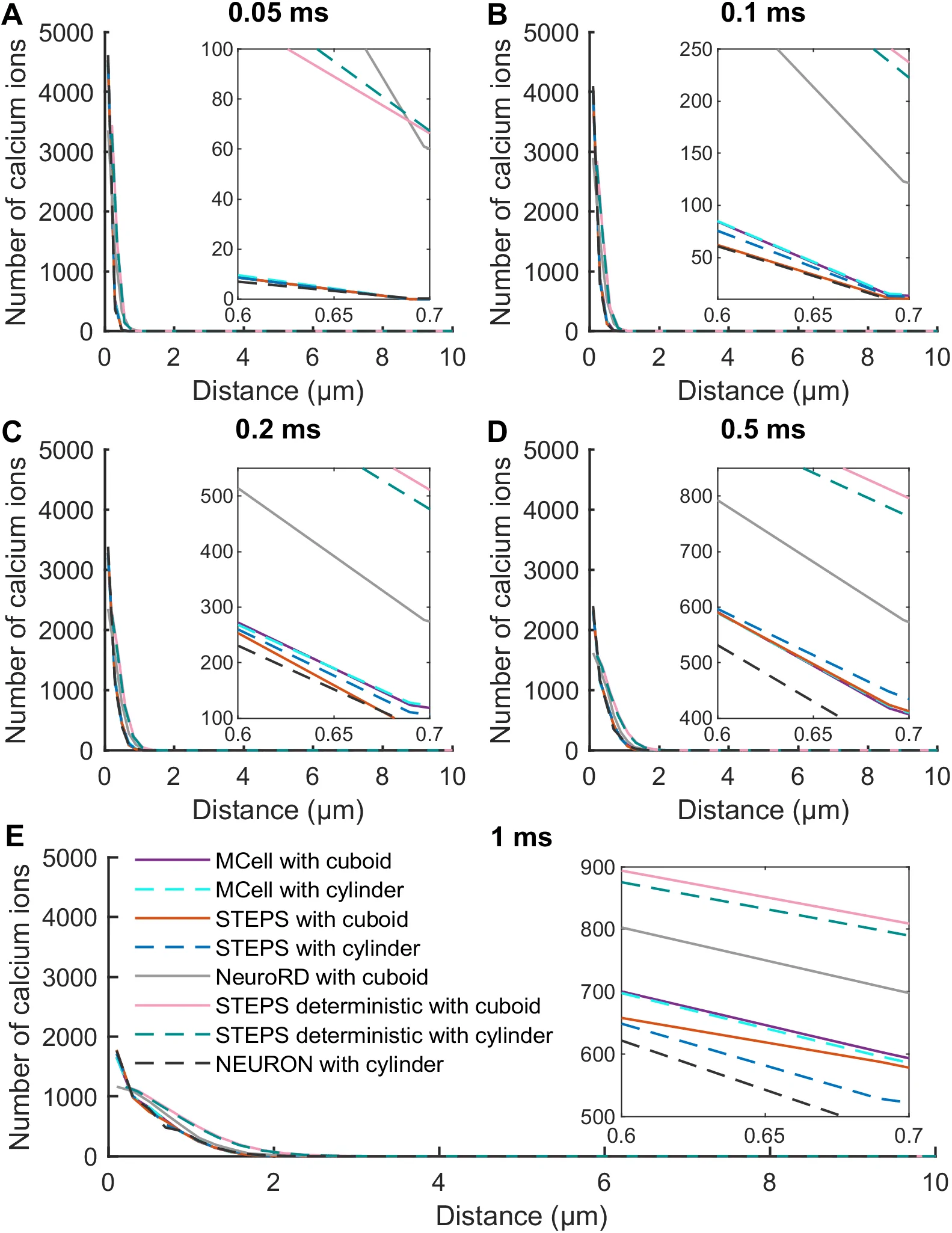
3D calcium diffusion results are similar in the simplified geometries with MCell, STEPS, NeuroRD, and NEURON rxd. MCell, STEPS, and NeuroRD simulations show a single realization of a stochastic run together with deterministic STEPS and NEURON simulations. Number of calcium ions per axial bin, plotted against axial distance from the injection point, at time points of **(A)** 0.05 ms, **(B)** 0.1 ms, **(C)** 0.2 ms, **(D)** 0.5 ms, and **(E)** 1 ms with insets.

**3D calcium diffusion in real cell morphologies.** Fig 5 presents 3D calcium diffusion results with 50 distance bins created with three suitable tools, MCell, STEPS, and NEURON, using the mouse Bergmann glial branchlet morphology 2 shown in Fig 1D [33]. For MCell and STEPS, the mean and standard deviation of 20 stochastic simulation runs are shown together with an example realization. The results produced by MCell and STEPS are highly consistent across all time points, indicating that the two simulators produce nearly identical diffusion behavior when operating on the same surface geometry. This agreement is expected because both simulators use the same OBJ-derived morphological surfaces and simulate diffusion within the same intracellular domain. Additionally, the deterministic STEPS simulation falls within the variation of the stochastic simulations. However, NEURON introduces some differences in the results, showing a faster diffusion from the injection site, especially in the last three time points (Fig 5C–5E). This difference likely arises from the different geometry representation used by NEURON. While MCell and STEPS operate directly on the original surface or volumetric meshes, NEURON simulations require converting the geometry into an SWC-based tree structure and subsequently discretizing the intracellular space into cubic voxels. This voxel-based representation may slightly smooth thin structures or introduce additional connectivity between nearby regions, which can reduce geometrical constraints on diffusion and result in somewhat faster spreading of calcium. The Bergmann glial branchlet morphology 2 (Fig 1D) contains complex structural features, including thin processes and locally looped regions that deviate from the tree-like organization typical of neuronal morphologies used in NEURON simulations (Fig 2D). Such features are difficult to represent exactly using an SWC-based skeleton and voxel discretization, which may lead to small deviations in the simulated diffusion dynamics. However, viewing the NRMSD presented in Table 6 and Pearson Correlation Coefficient presented in Table C in S1 Appendix for Fig 5, all tools are in close agreement. Similarly to Fig 5, Fig A in S1 Appendix shows consistent results for the mean and standard deviation of MCell and STEPS results for the mouse Bergmann glial branchlet morphology 1 [33] (Fig 1C). However, the diffusion is reaching a steady-state situation faster in Fig A in S1 Appendix than in Fig 5.

**Table 6:** Normalized root mean square deviation (NRMSD) calculated by dividing the RMSD by the range of values when comparing calcium diffusion simulation results between two simulation tools for astrocyte branchlet morphologies. Different time points 0.05 ms, 0.1 ms, 0.2 ms, 0.5 ms, and 1 ms represent subfigures A–E, respectively, plotted from calcium diffusion simulations in Figs 5–7. STEPS det and NEURON are deterministic simulations, others are stochastic simulations of which we used the mean of 20 simulations in the calculations. The NRMSD values are always below 5%.

Time point	Tools	Fig 5: Mouse BG 2	Fig 6: Mouse CX 1	Fig 7: Human CX 1
**0.05 ms**	**MCell & STEPS**	0.0043	0.13	0.16
	**MCell & NEURON**	0.0092	2.8	0.82
	**STEPS & NEURON**	0.0050	2.8	0.66
	**STEPS & STEPS det**	0.0004	0.067	0.030
	**STEPS det & NEURON**	0.0053	2.8	0.69
**0.1 ms**	**MCell & STEPS**	0.026	0.22	0.086
	**MCell & NEURON**	0.064	3.8	1.2
	**STEPS & NEURON**	0.039	3.6	1.1
	**STEPS & STEPS det**	0.017	0.12	0.052
	**STEPS det & NEURON**	0.055	3.7	1.2
**0.2 ms**	**MCell & STEPS**	0.014	0.17	0.11
	**MCell & NEURON**	0.11	4.1	1.7
	**STEPS & NEURON**	0.11	4.1	1.7
	**STEPS & STEPS det**	0.025	0.18	0.099
	**STEPS det & NEURON**	0.091	4.0	1.7
**0.5 ms**	**MCell & STEPS**	0.10	0.26	0.12
	**MCell & NEURON**	0.25	3.6	2.1
	**STEPS & NEURON**	0.21	3.5	2.0
	**STEPS & STEPS det**	0.056	0.23	0.10
	**STEPS det & NEURON**	0.22	3.4	2.0
**1 ms**	**MCell & STEPS**	0.11	0.36	0.22
	**MCell & NEURON**	0.61	3.5	2.4
	**STEPS & NEURON**	0.65	3.6	2.4
	**STEPS & STEPS det**	0.085	0.28	0.20
	**STEPS det & NEURON**	0.64	3.6	2.2

**Fig 5:**
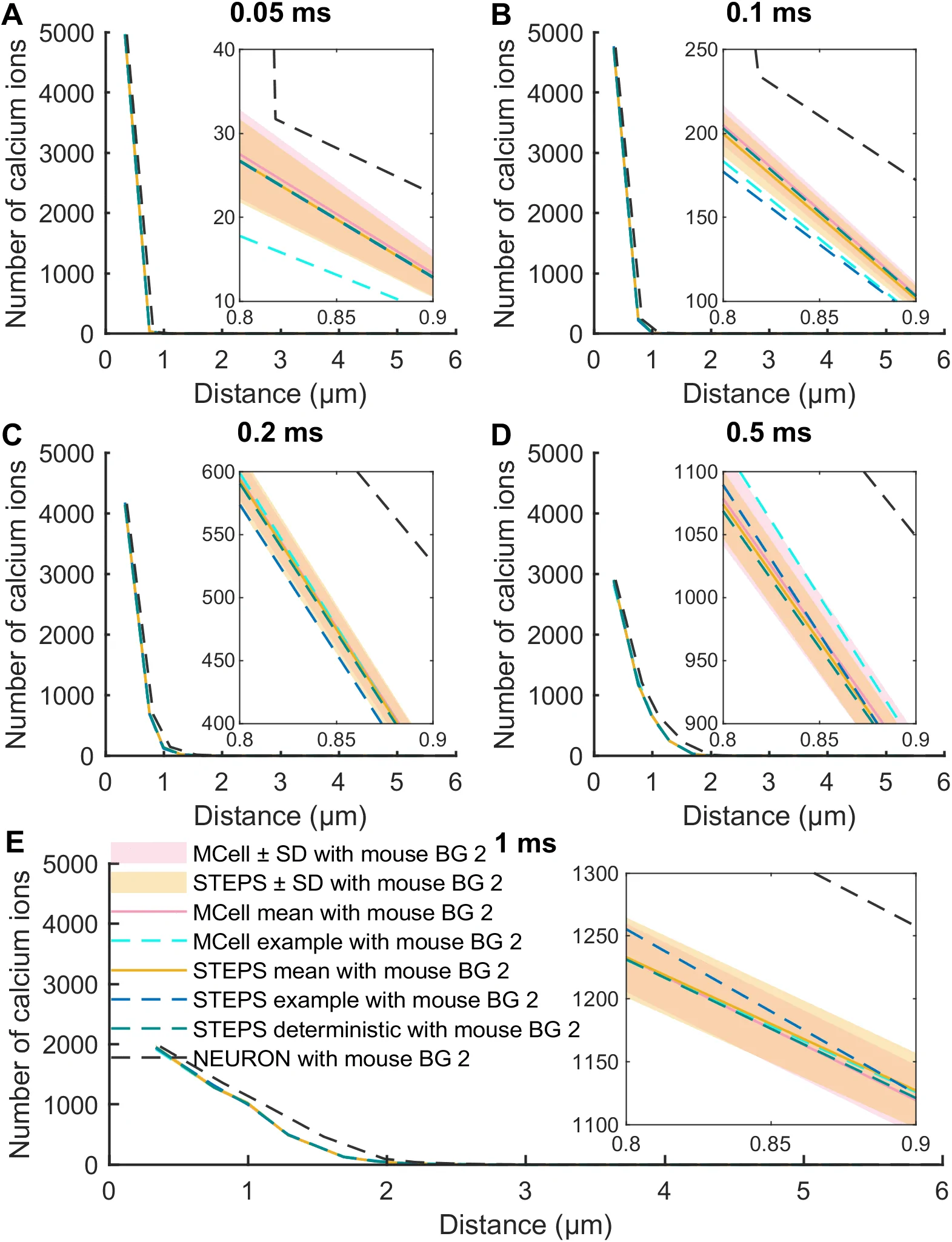
3D calcium diffusion simulations in the mouse Bergmann glial branchlet morphology 2 (Mouse BG 2) show similar results with MCell, STEPS, and NEURON rxd. Mean and standard deviation of 20 simulation runs shown with MCell and STEPS together with single realizations of MCell and STEPS runs and deterministic STEPS and NEURON simulations. Number of calcium ions plotted as a function of geodesic distance from the injection point at time points of **(A)** 0.05 ms, **(B)** 0.1 ms, **(C)** 0.2 ms, **(D)** 0.5 ms, and **(E)** 1 ms with insets. Shaded area indicates ±SD.

As in Fig 5, the 3D calcium diffusion results of MCell and STEPS show a close agreement throughout the simulation in Fig 6, which uses the mouse cortical astrocyte branchlet morphology 1 [31] (Fig 1E). In this case, the NEURON results are partially, throughout the simulation, landing more inside the standard deviation of the stochastic simulations of MCell and STEPS compared to the Bergmann glial branchlet morphology 2 shown in Fig 5. The cortical astrocyte branchlet morphology 1 contains fewer loops than Bergmann glial branchlet morphology 2 and exhibits a more tree-like organization of processes, resulting in lower geometrical complexity. Because NEURON was originally developed to represent neuronal morphologies as skeletal trees, the shape of the cortical astrocyte branchlet segment is computationally more compatible with the geometry representation used by the tool (Fig 2F). However, the NRMSD in Table 6 and Pearson Correlation Coefficient presented in Table C in S1 Appendix show closer agreement between the tools in Fig 5 than in Fig 6. The deterministic STEPS simulation agrees again more closely with the stochastic STEPS rather than the deterministic NEURON simulation. Fig B in S1 Appendix shows the simulation results for the mouse cortical astrocyte branchlet morphology 2 [31] (Fig 1F), which exhibits a behavior similar to that observed in Fig 6.

**Fig 6:**
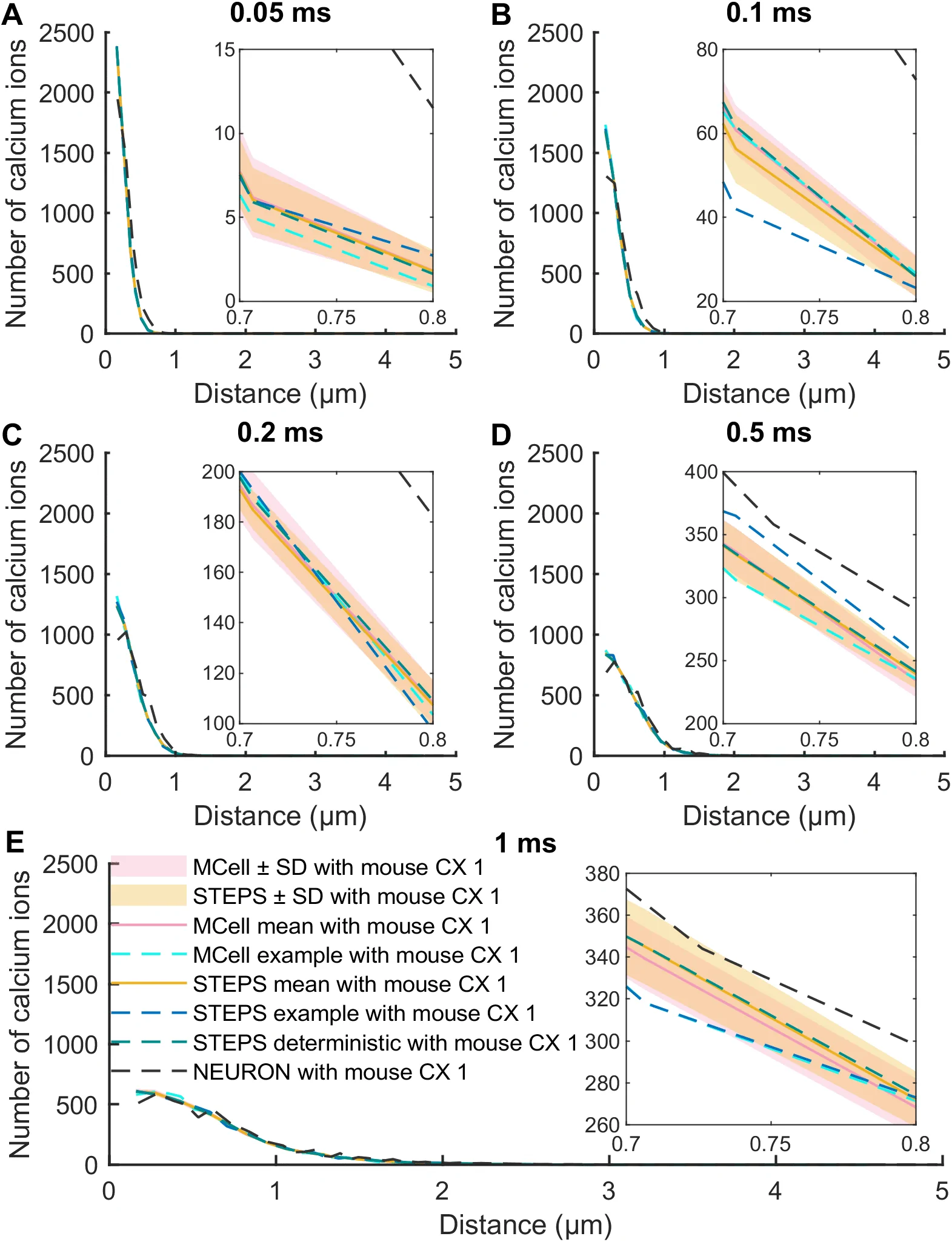
3D calcium diffusion simulations in the mouse cortical astrocyte branchlet morphology 1 (Mouse CX 1) show similar results with MCell, STEPS, and NEURON rxd. Mean and standard deviation of 20 simulation runs shown with MCell and STEPS together with single realizations of MCell and STEPS runs and deterministic STEPS and NEURON simulations. Number of calcium ions plotted as a function of geodesic distance from the injection point at time points of **(A)** 0.05 ms, **(B)** 0.1 ms, **(C)** 0.2 ms, **(D)** 0.5 ms, and **(E)** 1 ms with an inset. Shaded area indicates ±SD.

Similarly to Figs 5 and 6, the mean and standard deviation of the results with the human cortical astrocyte branchlet morphology 1 [32] (Fig 1G) in MCell and STEPS are consistent, as presented in Fig 7, Table 6, and Table C in S1 Appendix. However, NEURON introduces differences in the results compared to the other tools that use stochastic and deterministic solvers, showing faster diffusion from the injection site. This behavior may arise in part from the voxel-based discretization used in NEURON, where thin constrictions in the original morphology can become slightly smoothed in the cubic grid representation (Fig 2J). Morphologies such as the mouse Bergmann glial branchlets and the human cortical astrocyte branchlets appear to contain more narrow passages around the injection region than the mouse cortical astrocyte branchlet morphology 1, which may increase local diffusion barriers and lead to slightly slower spread of calcium ions in the NEURON simulations. Fig C in S1 Appendix shows a similar behavior for the human cortical astrocyte branchlet morphology 2 [32] (Fig 1H) compared to Fig 7, except that diffusion occurs faster in Fig 7.

**Fig 7:**
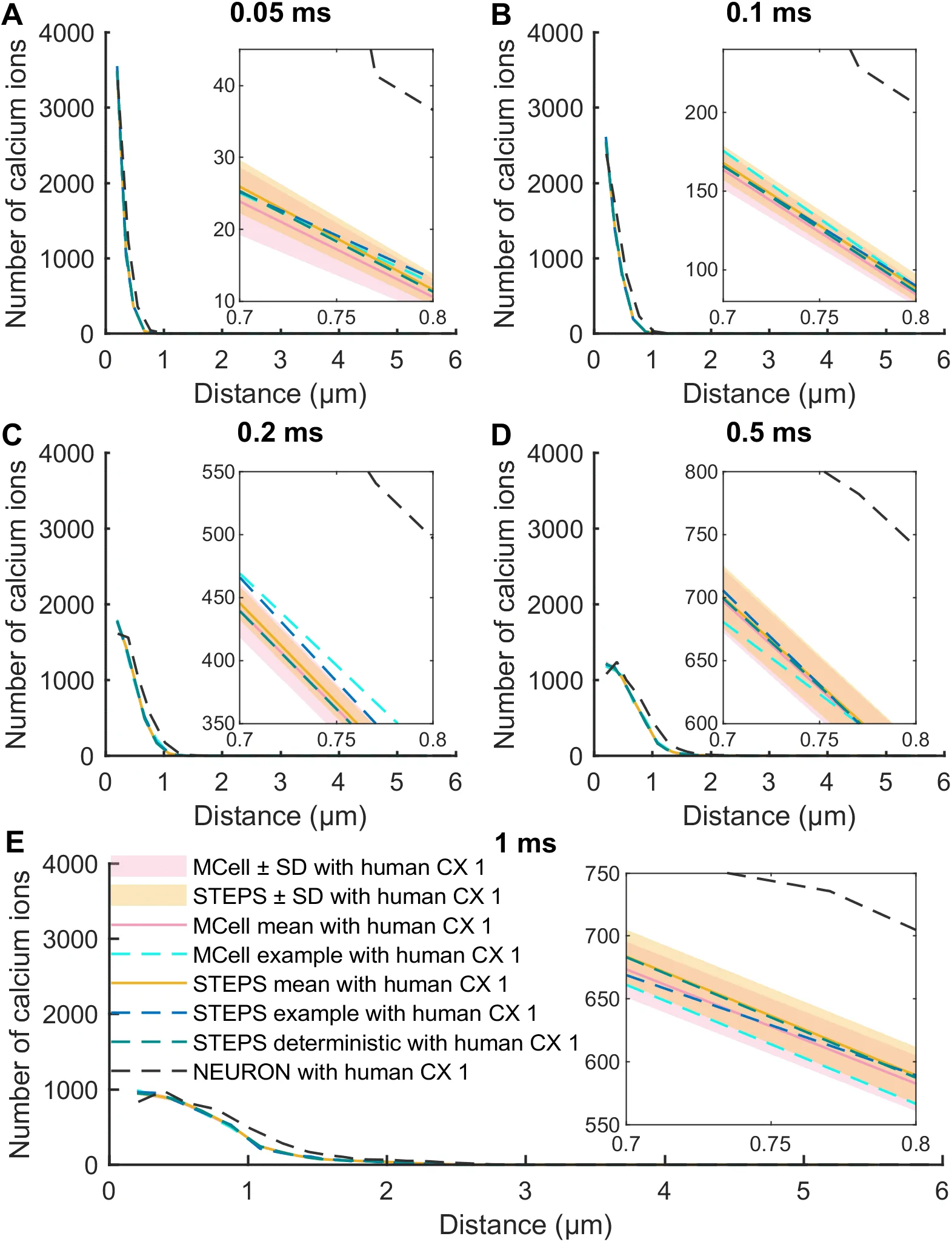
3D calcium diffusion simulations in the human cortical astrocyte branchlet morphology 1 (Human CX 1) show similar results with MCell, STEPS, and NEURON rxd. Mean and standard deviation of 20 simulation runs shown with MCell and STEPS together with single realizations of MCell and STEPS runs and deterministic STEPS and NEURON simulations. Number of calcium ions plotted as a function of geodesic distance from the injection point at time points of **(A)** 0.05 ms, **(B)** 0.1 ms, **(C)** 0.2 ms, **(D)** 0.5 ms, and **(E)** 1 ms with an inset. Shaded area indicates ±SD.

Fig 8 and Fig D in S1 Appendix present similar morphological representations of calcium diffusion simulations with MCell, STEPS, and NEURON at a time point of 1 ms in the branchlets of mouse Bergmann glial morphologies [33], mouse cortical astrocyte morphologies [31], and human cortical astrocyte morphologies [32] together with the STEPS representation of the initial time point showcasing the starting point of the calcium injection. In general, the spatial distribution patterns of calcium ions are visually similar across the three simulators, indicating consistent diffusion behavior despite the different geometry representations used by the tools. The visualizations also highlight how calcium diffusion is constrained by the underlying branchlet geometry, with the spread following the morphology rather than forming a uniform distribution before the end of the simulation. Any differences between the simulations appear subtle on this time scale, but are consistent with the quantitative trends observed in Figs 5–7, Table 6, and Figs A–C and Tables B and C in S1 Appendix.

**Fig 8:**
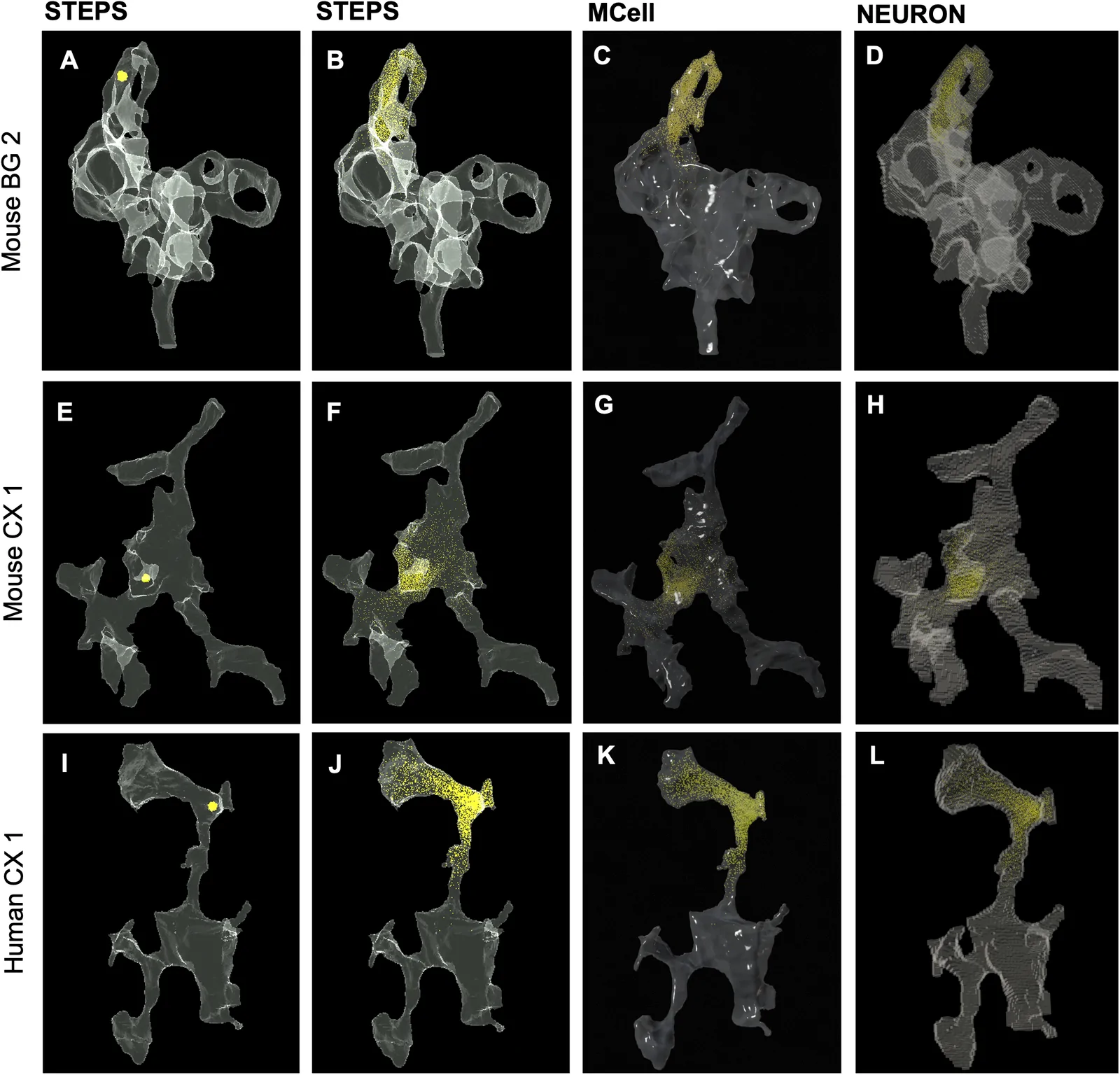
Morphological presentation of the calcium diffusion simulations in astrocyte branchlet morphologies shows similar results with MCell, STEPS, and NEURON. **(A, E,** **I)** The initial time point in the beginning of the STEPS simulations for the branchlets of the mouse Bergmann glial morphology 2 (Mouse BG 2) [33], mouse cortical astrocyte morphology 1 (Mouse CX 1) [31], and human cortical astrocyte morphology 1 (Human CX 1) [32]. **(B, F,** **J)** Stochastic STEPS simulations at 1 ms. **(C, G,** **K)** MCell simulations at 1 ms. **(D, H,** **L)** NEURON simulations at 1 ms. Calcium ions are illustrated in yellow. For visualization of the NEURON simulations in this figure, voxels were rendered as uniform cuboids of edge length dx centered at voxel nodes to improve visual clarity.

#### Reaction–diffusion simulations.

In this section, the results are presented as the amount of calcium, calmodulin without calcium bound (CaM, see Table 5), and calmodulin with four calcium ions bound (Ca_4_CaM, see Ca_4_CaM_N2C2_ in Table 5) as a function of time. Calmodulin is evenly distributed at the beginning of the simulation through the geometries and calcium injections are performed in the same way as in diffusion simulations.

**Reaction–diffusion simulations with 1D diffusion in simplified geometries.** Fig 9 presents reaction–diffusion simulation results with 1D diffusion obtained from NeuroRD and NEURON simulations. The results between NEURON and NeuroRD are again highly consistent, but the stochastic nature of the NeuroRD reaction–diffusion mechanisms is visible in Fig 9.

**Fig 9:**
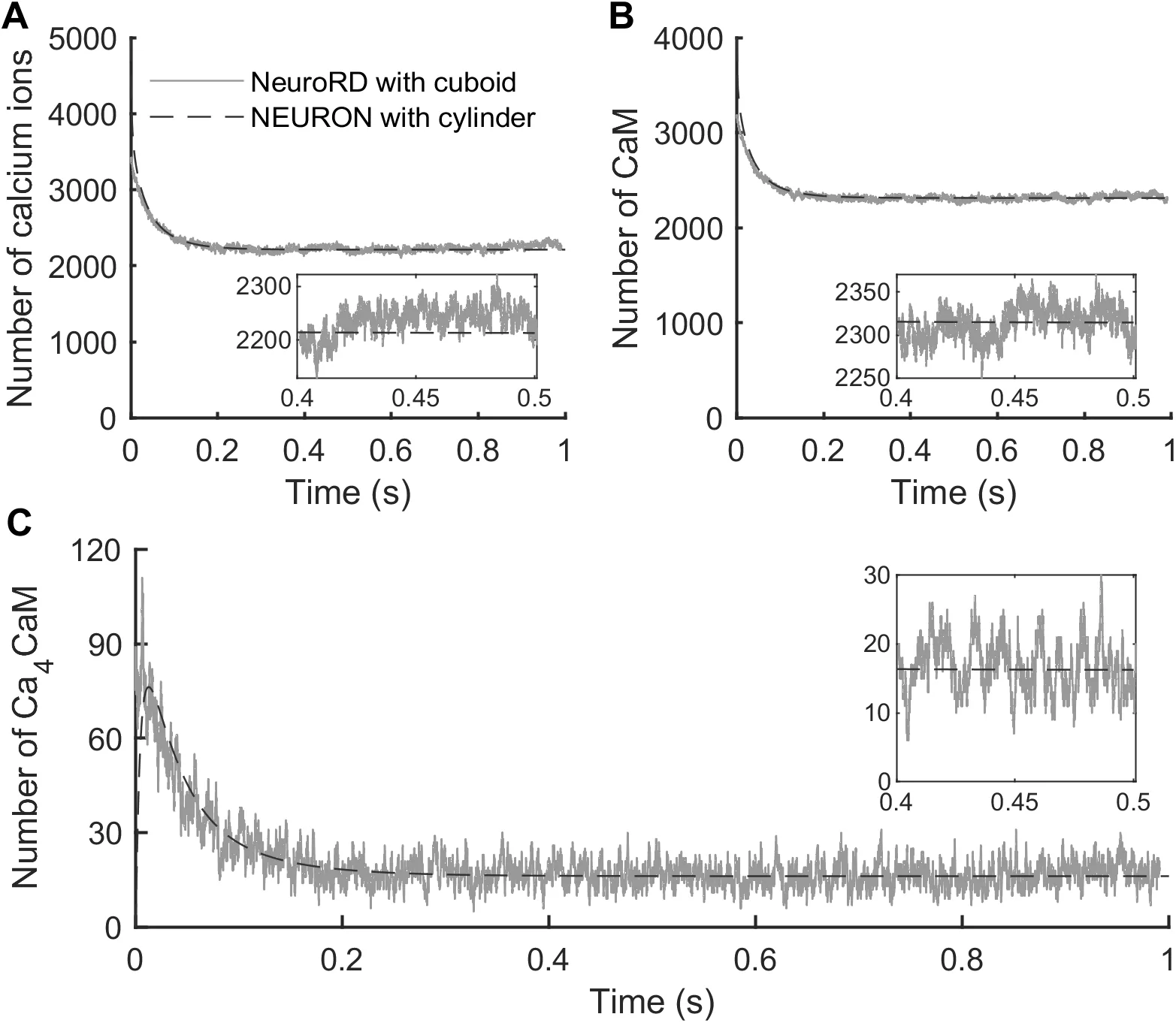
Reaction–diffusion simulations with 1D diffusion show highly consistent results across NeuroRD and NEURON rxd in the simplified geometries with 25 subvolumes. NeuroRD simulation shows a single realization of a stochastic run together with deterministic NEURON simulation. Number of **(A)** calcium ions, **(B)** calmodulin (CaM) molecules, and **(C)** Ca_4_CaM complexes plotted as a function of time with insets.

**Reaction–diffusion simulations with 3D diffusion in simplified geometries.** Fig 10 presents reaction–diffusion simulation results with 3D diffusion with MCell, STEPS, NeuroRD, and NEURON, using cuboid and cylinder morphologies shown in Fig 1A and 1B. The results across all tools are highly consistent. This close agreement is expected because the cuboid and cylindrical geometries are simple and regular structures that can be represented accurately by all simulators without complex morphology conversions, showing only slight variations due to the use of stochastic solvers (STEPS, MCell, and NeuroRD) compared to a deterministic solver (NEURON). The number of Ca_4_CaM molecules peaks around 90–100 for simulations in STEPS and MCell, while in NeuroRD and NEURON the peak falls slightly below 90 and 80, respectively. We were unable to run the deterministic reaction–diffusion solver provided by STEPS (TetODE) on the original, finely tetrahedralized cylinder mesh within a practical computational time. To assess the agreement between the deterministic and stochastic approaches, we generated a coarser tetrahedral representation of the cylinder while retaining its overall geometrical characteristics (Fig F in S1 Appendix) and simulated the same reaction–diffusion model using both TetODE and Tetexact. Under these matched conditions, the deterministic and stochastic STEPS simulations showed close agreement in the resulting dynamics (Fig G in S1 Appendix).

**Fig 10:**
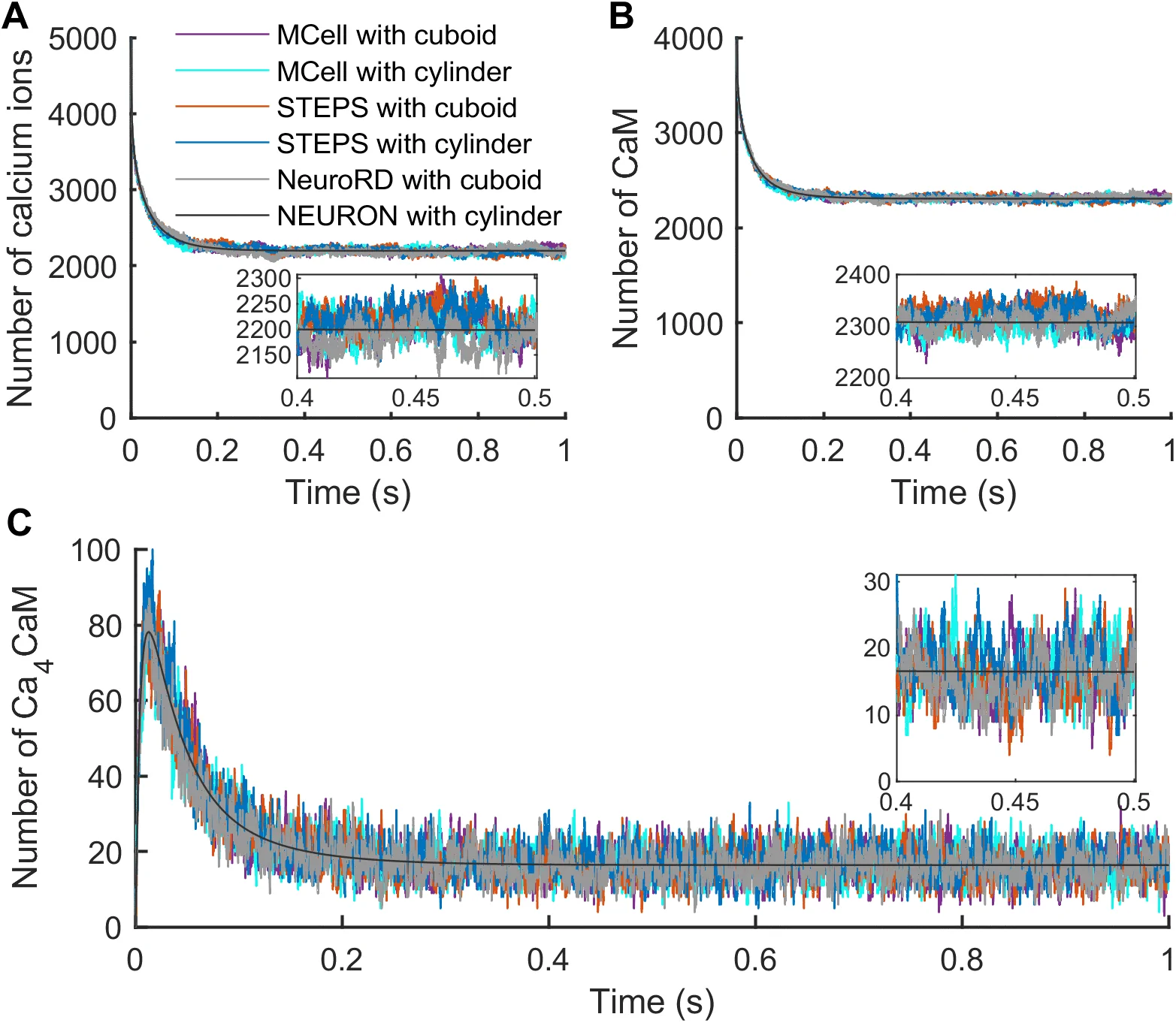
Reaction–diffusion simulations with 3D diffusion in the simplified geometries show highly consistent results with MCell, STEPS, NeuroRD, and NEURON rxd. MCell, STEPS, and NeuroRD simulations show a single realization of a stochastic run together with deterministic NEURON simulation. Number of **(A)** calcium ions, **(B)** calmodulin (CaM) molecules, and **(C)** Ca_4_CaM complexes plotted as a function of time with insets.

**Reaction–diffusion simulations with 3D diffusion in real cell morphologies.** Fig 11 presents reaction–diffusion simulations with 3D diffusion with three suitable tools, MCell, STEPS, and NEURON, using two Bergmann glial branchlet morphologies shown in Fig 1C and 1D [33]. In Fig 11A–11C, we show a single realization of MCell and STEPS simulations for both Bergmann glial branchlet morphologies together with NEURON simulations. For each morphology, the performance of the tools is highly consistent, although in NEURON, calcium and calmodulin seem to plateau at a slightly higher level with Bergmann glial branchlet morphology 1, leading to a slightly lower baseline of Ca_4_CaM. In Fig 11D, we show the mean and standard deviation of 20 stochastic runs of MCell and STEPS (shaded area indicates ±SD) together with NEURON simulation for the Bergmann glial branchlet morphology 2. It can be seen that, despite the stochastic nature of the tools, the variability between the 20 simulation runs remains relatively small. This reflects the large number of molecules involved in the reactions, which reduces the relative magnitude of stochastic fluctuations. In addition, the mean trajectories obtained with MCell and STEPS and the NRMSD values presented in Table 7 and Pearson Correlation Coefficient values presented in Table E in S1 Appendix are very similar, indicating consistent reaction–diffusion dynamics between the two simulation tools and with NEURON.

**Table 7:** Normalized root mean square deviation (NRMSD) calculated by dividing the RMSD by the range of values when comparing reaction–diffusion simulation results between two simulation tools for astrocyte branchlet morphologies. Calcium, calmodulin, and Ca_4_CaM present the model variables obtained from calcium–calmodulin reaction–diffusion simulations in Figs 11–13. NEURON uses deterministic simulations, others are stochastic simulations of which we used the mean of 20 simulations in the calculations. The NRMSD values are always below 8%.

Model variable	Tools	Fig 11: Mouse BG 2	Fig 12: Mouse CX 1	Fig 13: Human CX 1
**Calcium**	**MCell & STEPS**	0.6004	0.2689	0.3817
	**MCell & NEURON**	0.7142	4.4597	0.9643
	**STEPS & NEURON**	0.6872	4.4768	0.8595
**Calmodulin**	**MCell & STEPS**	0.5889	0.2837	0.3507
	**MCell & NEURON**	0.6508	3.4544	0.6147
	**STEPS & NEURON**	0.6678	3.4938	0.6602
**Ca_4_CaM**	**MCell & STEPS**	2.0446	2.6098	1.6774
	**MCell & NEURON**	1.5453	7.9362	3.3505
	**STEPS & NEURON**	1.8070	7.4053	2.7452

**Fig 11:**
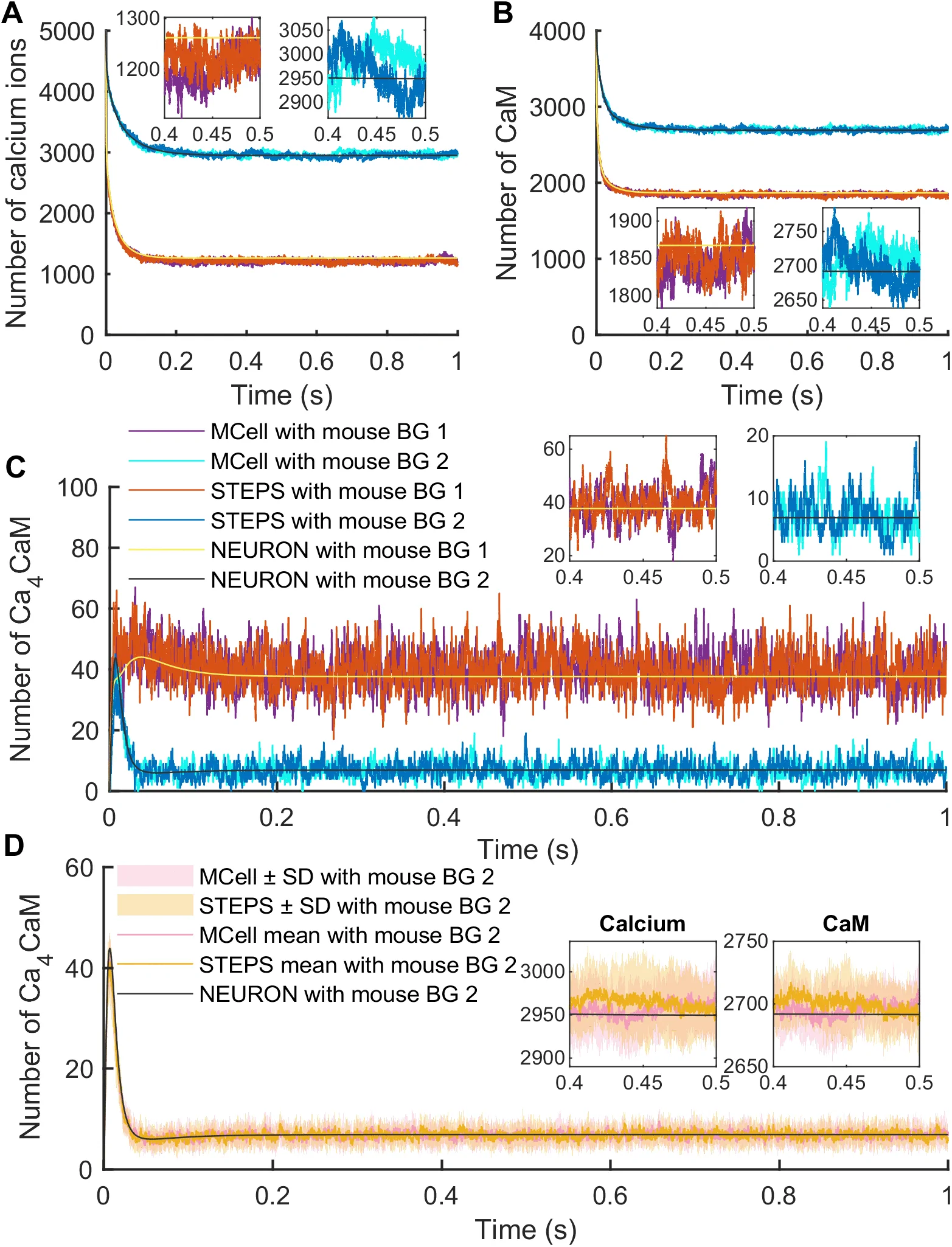
Reaction–diffusion simulations with 3D diffusion in the two mouse Bergmann glial branchlet morphologies (Mouse BG 1 and 2) show highly consistent results with MCell, STEPS, and NEURON rxd. Number of **(A)** calcium ions, **(B)** calmodulin (CaM) molecules, and **(C)** Ca_4_CaM complexes plotted as a function of time from a single realization of a stochastic run with MCell and STEPS together with deterministic NEURON simulations in both of the morphologies, and **(D)** mean and standard deviation (shaded area indicates ±SD) of Ca_4_CaM from 20 MCell and STEPS simulation runs together with NEURON simulation with the mouse Bergmann glial branchlet morphology 2 plotted as a function of time with insets for calcium and calmodulin.

Fig 12 presents reaction–diffusion simulations with 3D diffusion with three suitable tools, MCell, STEPS, and NEURON, using two mouse cortical astrocyte branchlets shown in Fig 1E and 1F [31]. The results highlight otherwise consistent behavior, with a slight difference when analyzing the NEURON mouse cortical astrocyte branchlet morphology 1 results with the other tools. The calcium and calmodulin baseline stays slightly higher with NEURON, reflecting also a smaller peak in Ca_4_CaM formation. Whereas in MCell and STEPS Ca_4_CaM reaches around 90 molecules in the beginning of the simulation with mouse cortical astrocyte branchlet morphology 1 in Fig 12C and around 80 in Fig 12D, for NEURON the peak sets around 50 molecules (Fig 12C and 12D). Table 7 and Table E in S1 Appendix also confirm that the different tools provide consistent results, except for Ca_4_CaM, where the NRMSD between the NEURON simulation and the stochastic tools exceeds 7%. As we were unable to simulate the original astrocyte branchlet morphologies with the deterministic reaction–diffusion algorithm provided by STEPS, we simplified the mouse cortical astrocyte branchlet morphology 1 (Fig F in S1 Appendix) and show in Fig H in S1 Appendix that the deterministic and stochastic simulation results of STEPS are consistent.

**Fig 12:**
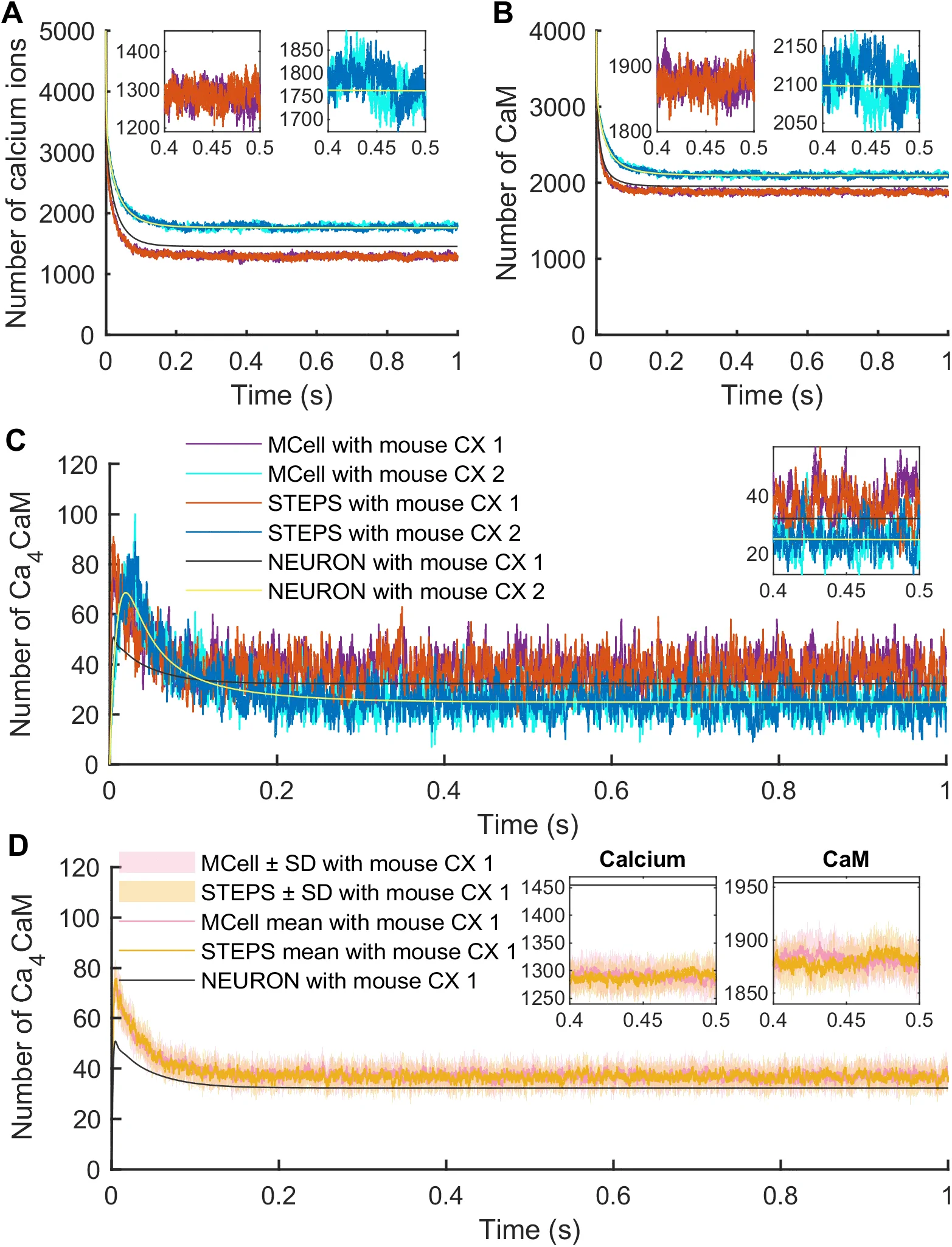
Reaction–diffusion simulations with 3D diffusion in the two mouse cortical astrocyte branchlet morphologies (Mouse CX 1 and 2) show consistent results with MCell, STEPS, and NEURON rxd. Number of **(A)** calcium ions, **(B)** calmodulin (CaM) molecules, and **(C)** Ca_4_CaM complexes plotted as a function of time from a single realization of a stochastic run with MCell and STEPS together with deterministic NEURON simulations in both of the morphologies, and **(D)** mean and standard deviation (shaded area indicates ±SD) of Ca_4_CaM from 20 MCell and STEPS simulation runs together with NEURON simulation with the mouse cortical astrocyte branchlet morphology 1 plotted as a function of time with insets for calcium and calmodulin.

Fig 13 presents reaction–diffusion simulations with 3D diffusion with three suitable tools, MCell, STEPS, and NEURON, using two branchlets of human cortical astrocytes shown in Fig 1G and 1H [32]. For both human cortical astrocyte branchlet morphologies, NEURON follows the corresponding STEPS and MCell results, although the degradation of Ca_4_CaM is slightly faster. Similarly to the mouse cortical astrocyte branchlet simulation, the Ca_4_CaM peak in the NEURON human cortical astrocyte branchlet morphology 1 is about 100 molecules and does not reach as high as the peaks observed in the MCell and STEPS simulations, which are about 140 molecules in Fig 13C and 120–125 in Fig 13D. However, for human cortical astrocyte branchlet morphology 2, also NEURON can be considered to follow the Ca_4_CaM curve obtained with STEPS and MCell. The NRMSD values in Table 7 and Pearson Correlation Coefficient values in Table E in S1 Appendix confirm that different tools provide consistent results for human cortical astrocyte branchlet morphology 1.

**Fig 13:**
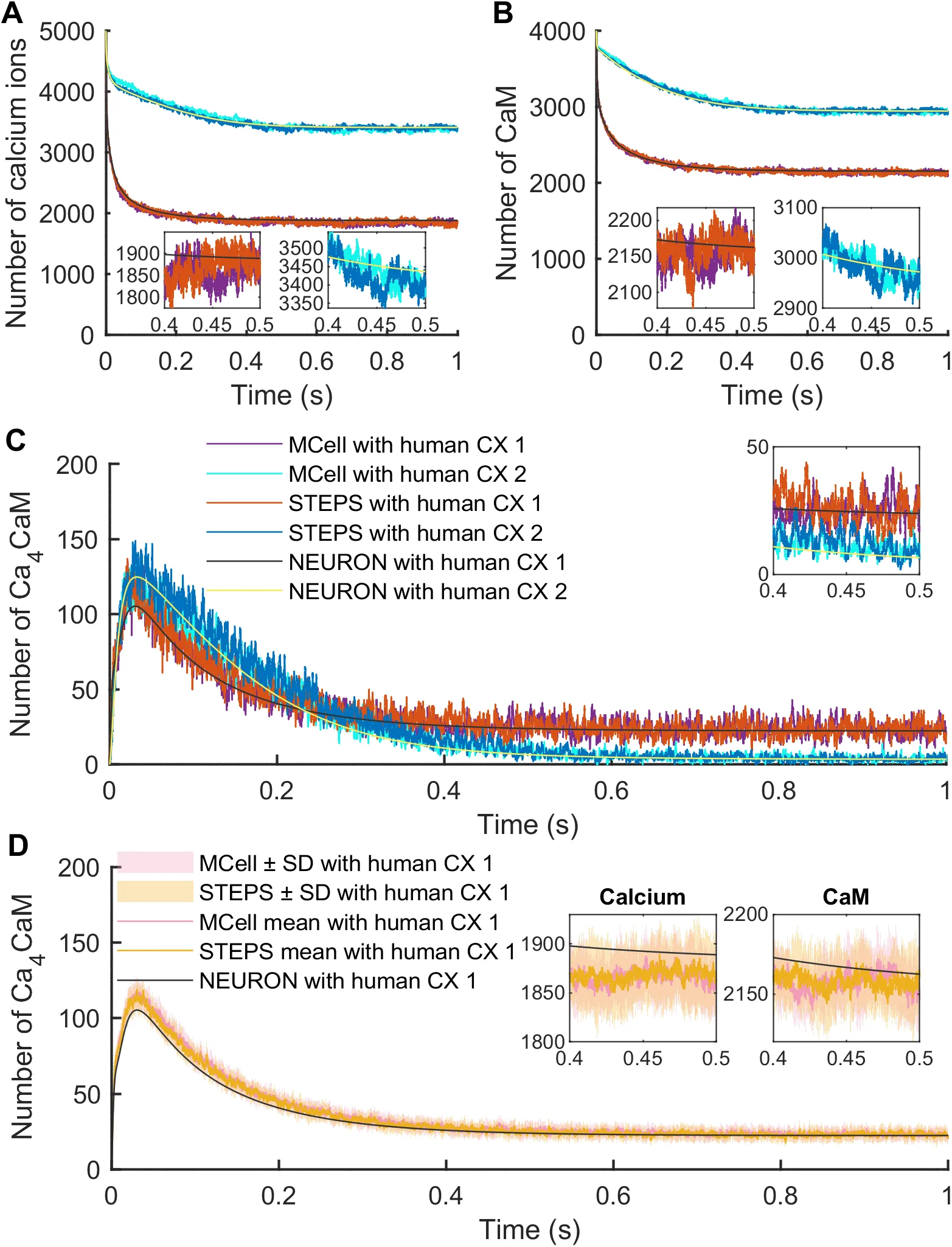
Reaction–diffusion simulations with 3D diffusion in the two human cortical astrocyte branchlet morphologies (Human CX 1 and 2) show consistent results with MCell, STEPS, and NEURON rxd. Number of **(A)** calcium ions, **(B)** calmodulin (CaM) molecules, and **(C)** Ca_4_CaM complexes plotted as a function of time from a single realization of a stochastic run with MCell and STEPS together with deterministic NEURON simulations in both of the morphologies, and **(D)** mean and standard deviation (shaded area indicates ±SD) of Ca_4_CaM from 20 MCell and STEPS simulation runs together with NEURON simulation with the human cortical astrocyte branchlet morphology 1 plotted as a function of time with insets for calcium and calmodulin.

Fig 14 and Fig E in S1 Appendix present morphological representations of reaction–diffusion simulations in the branchlets of mouse Bergmann glial morphologies [33], mouse cortical astrocyte morphologies [31], and human cortical astrocyte morphologies [32] with MCell, STEPS, and NEURON. The visualization of the reaction–diffusion simulations indicates a consistent reaction–diffusion behavior for calcium, calmodulin, and Ca_4_CaM despite the different geometric representations used by the tools. However, a slightly broader spread of Ca_4_CaM can be observed in the MCell simulations for Bergmann glial branchlet morphology 2 than in the other tools (Fig 14B–14D). Fig E in S1 Appendix shows a smaller number of Ca_4_CaM molecules formed when using the human cortical astrocyte branchlet morphology 2 with all tools compared to other morphologies.

**Fig 14:**
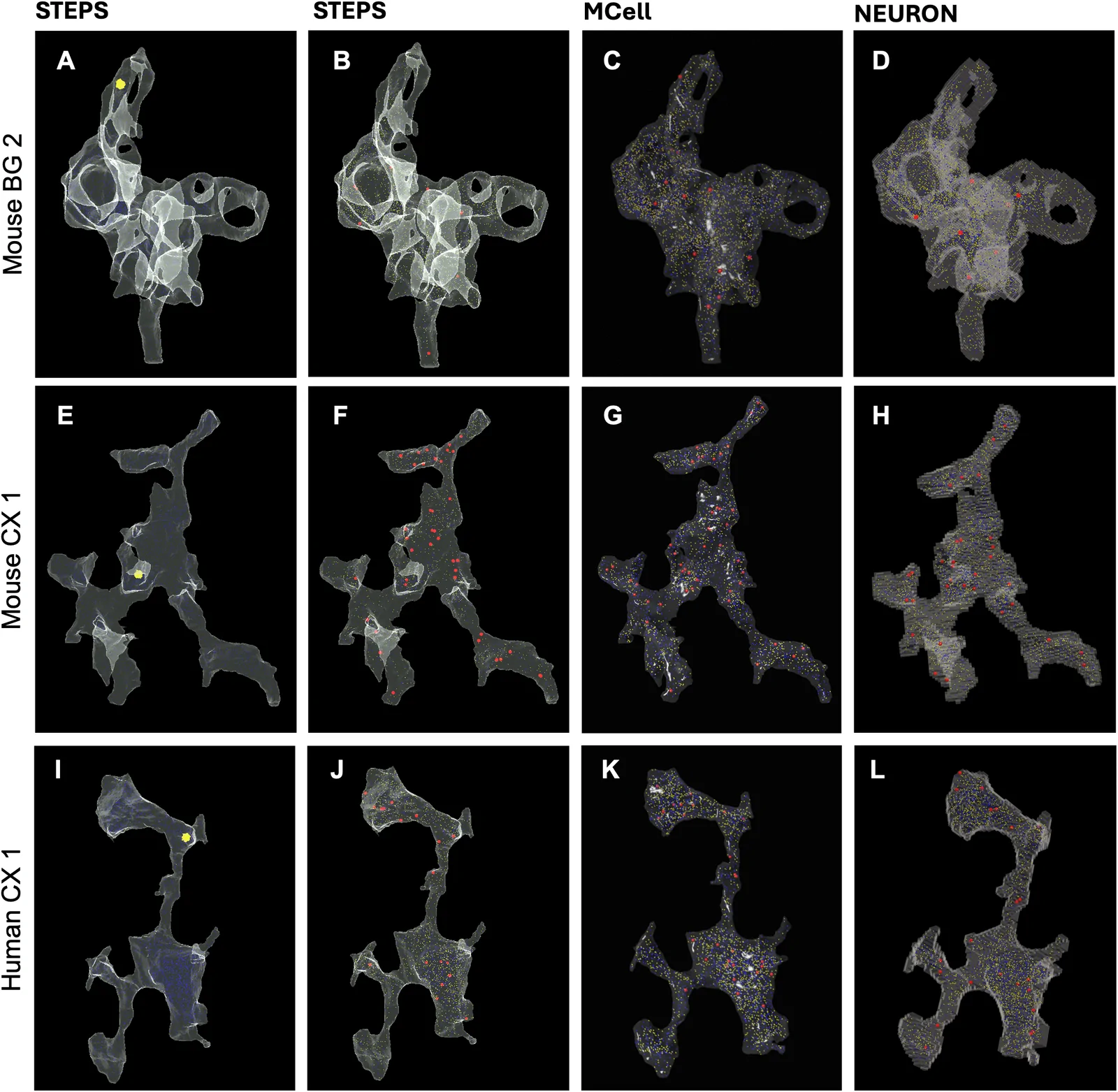
Morphological presentation of the reaction–diffusion simulations in astrocyte branchlet morphologies shows similar results with MCell, STEPS, and NEURON. **(A, E,** **I)** The initial time point in the beginning of the STEPS simulations for the branchlets of the mouse Bergmann glial morphology 2 (Mouse BG 2) [33], mouse cortical astrocyte morphology 1 (Mouse CX 1) [31], and human cortical astrocyte morphology 1 (Human CX 1) [32]. **(B, F,** **J)** Stochastic STEPS simulations at 1 s. **(C, G,** **K)** MCell simulations at 1 s. **(D, H,** **L)** NEURON simulations at 1 s. Calcium ions are illustrated in yellow, calmodulin in blue, and Ca_4_CaM in red. For visualization of the NEURON simulations in this figure, voxels were rendered as uniform cuboids of edge length dx centered at voxel nodes to improve visual clarity.

### Applicability of the reaction–diffusion frameworks to simplified and astrocyte geometries

In terms of general usability and suitability, we assessed the implementation process for the selected morphologies, diffusion characteristics, and chemical reactions. In addition, we present our views on the advantages and limitations of the tools.

With the CellBlender interface for MCell, model building, such as adding molecules, reactions, release sites, and model objects (e.g., OBJ morphology files), is relatively easy due to intuitive interface menus. The default menus also offer explanations and clarify the units used, reducing the need for manuals when implementing models. Although the documentation and tutorials provided on the MCell website and in [67] cover the information needed to design and run simulations, the information is often outdated, as they describe MCell3 functionalities with Blender version 2.79 and not with MCell4 with Blender version 2.93. As an example, the documentation describes trimolecular functions that are available for MCell3 but not for MCell4. A Python interface is available for MCell4, with guidance for model creation and execution [13]. This Python interface was not used in this study, but it could provide additional flexibility for more complex customizations. In addition, MCell makes it complicated to track the diffusion distance, but such can be done with custom scripts. One of the advantages of MCell and CellBlender is the ability to use real cell morphologies, such as the morphologies we used in this study [31–33]. Any “watertight” 3D mesh with triangular non-overlapping faces can be utilized in the simulations. We recommend designing complex meshes in Blender and then importing them to CellBlender and MCell. Another usability advantage is the ability to implement, simulate, and visualize all simulations in one GUI with Blender/CellBlender. Also, the data are easily exported to be further analyzed and visualized, for example, in Python with your own scripts.

The use of STEPS was straightforward through its Python interface, which allows flexible implementation of reaction–diffusion models directly in code. The STEPS website provides a comprehensive manual and tutorials that were sufficient to implement the models used in this study. A major advantage of STEPS is its ability to operate directly on tetrahedral volumetric meshes, allowing physically accurate representation of complex intracellular geometries. This makes it particularly suitable for simulations in highly irregular morphologies, such as astrocyte branchlets. However, the generation, quality control, and optimization of tetrahedral meshes require additional preprocessing steps compared to surface-based approaches. When saving spatiotemporal simulation data, additional post-processing was required to extract spatial profiles from the tetrahedral mesh. Per-tetrahedron molecule counts were aggregated into iso-volume bins defined along the geodesic distance within the morphology, enabling direct comparison of spatial calcium distributions across simulations. STEPS also provides convenient built-in visualization tools through the steps.visual interface, which enable interactive inspection of simulation results during runtime. These tools allow visualization of molecule counts, concentrations, and spatial distributions directly on the tetrahedral mesh, as well as time-series plots of selected quantities. This functionality facilitates rapid inspection, debugging, and qualitative analysis of simulations without requiring external visualization software.

The NeuroRD simulator does not, in general, support 1D simulations, but a 1D simulation can be forced using a two-dimensional (2D) simulation and manipulating the segmentation of the morphology. NeuroRD divides the 2D morphology into squares or rectangles where the maximal width and length of each rectangle is defined using the defaultMaxElementSide parameter. After defining the morphology as a sequence of squares, we used the length of these squares as the defaultMaxElementSide parameter. This prevented the simulator from dissecting the squares we defined in the morphology file in the y-direction and thus kept the representation of the morphology in 1D. For the 3D cuboid morphology, we determined the defaultMaxElementSide so that the segments could be organized in a 3D representation within the volume of the geometry. After creating the 1D and 3D implementations, running the simulations and visualizing the results in Python were straightforward. However, implementing complex 3D structures in NeuroRD can be more challenging, especially without automated tools. Here, the goal was not to introduce new functionality but to examine the existing capabilities of the tools; therefore, realistic morphologies were not implemented in NeuroRD.

Similarly to STEPS, the use of NEURON was relatively user-friendly through its Python interface and the rxd module, which enables the implementation of diffusion and reaction–diffusion models directly in code. Comprehensive documentation and tutorials are available on the NEURON website, although some sections seemed partially unfinished. When scripting with Python, diffusion processes and chemical reactions were easy to define, and simulation outputs such as molecule and ion counts could be readily analyzed as functions of spatial distance and time. Simulation data and figures could also be easily exported for further analysis. Morphological visualization of spatial simulation results typically required exporting data to external tools such as ParaView, although NEURON itself provides convenient plotting utilities for inspecting time series and other model variables.

Compared to simulators that operate directly on volumetric meshes, realistic morphologies required additional preprocessing steps in NEURON because geometries must be represented as tree-like SWC structures. After reconstruction and volume scaling (see Methods), the resulting morphologies reproduced the original mesh volumes with deviations of approximately 1–6% for most geometries and approximately 17% for mouse cortical astrocyte branchlet morphology 1 (Table 2). The corresponding surface-to-volume ratios remained comparable between the different simulator representations, indicating that the geometry conversion preserved the overall morphological characteristics reasonably well. The morphometric analysis also highlights the geometric differences between simplified and realistic morphologies. The cuboid and cylindrical geometries exhibited surface-to-volume ratios of approximately 9–10 μm^-1^, while the astrocyte branchlet morphologies showed substantially higher values ranging from approximately 9–20 μm^-1^ (Table 2). These higher surface-to-volume ratios reflect the thin and elongated structure of astrocytic processes, where a relatively large membrane surface surrounds a small cytosolic volume. Such geometrical characteristics are expected to influence intracellular diffusion and reaction dynamics, particularly in narrow branchlets where spatial confinement becomes significant.

An important distinction between 1D and 3D implementations in NEURON concerns the representation of complex topology. In the traditional 1D cable formalism, morphologies must be represented as tree-like structures without loops, which limits the ability to faithfully capture the highly interconnected architecture characteristic of astrocytic processes. In contrast, NEURON 3D rxd operates on a volumetric voxel grid constructed from the underlying geometry, allowing spatially adjacent structures to be connected through shared voxels. As a result, closed loops and locally fused processes present in astrocyte morphologies can be represented in NEURON 3D rxd but not in the 1D cable framework. In general, NEURON provides a flexible and accessible environment for implementing reaction–diffusion models using Python. However, the need for morphology conversion and external visualization tools introduces additional preprocessing steps compared to simulators that operate directly on volumetric meshes.

## Discussion

In this study, we analyze four state-of-the-art, open-access simulation tools used primarily in the modeling of neuronal reaction–diffusion systems: MCell, STEPS, NeuroRD, and the NEURON rxd module (Table 1). In addition, we examine astrocyte branchlet morphologies and their implications for reaction–diffusion modeling. We found that the structural heterogeneity of human astrocyte [32] and mouse Bergmann glial branchlets [33] gives rise to qualitatively distinct calcium signaling regimes, demonstrating that fine-scale morphology critically shapes astrocytic dynamics. Throughout the simulators, the key model variables, namely the amounts of calcium, calmodulin, and Ca_4_CaM, exhibit markedly different signaling behaviors depending on the local geometry (Figs 11 and 13). These results highlight the strong sensitivity of the astrocytic calcium dynamics to microstructural features, such as narrow passages, complex branching patterns, and loop-like structures. Importantly, these findings place strong requirements on simulation frameworks, which must accurately resolve morphology-dependent diffusion and reaction processes to capture biologically meaningful dynamics.

The four open-access tools were selected because, to the best of our knowledge, they have not been jointly examined in a single study [17,18,27,68–70], particularly in the context of realistic astrocyte morphologies. Our motivation was to assess their suitability for future studies of calcium dynamics in complex astroglial branchlets, including mouse and human cortical astrocyte branchlets [31,32], as well as Bergmann glial branchlets [33] (Figs 1 and 2 and Tables 2 and 3). Although individual tools and subsets of them have been analyzed previously [28,29], and some pairwise computational comparisons exist (e.g., [17,18,27]), a systematic analysis across these four tools in both simplified and realistic 3D geometries remains lacking. Here, we provide a practical analysis of the tools with respect to their usability, scope, and resulting simulation outputs. Our goal was not to produce fully optimized or tool-specific benchmark implementations but rather to develop consistent and comparable simulation setups across the different platforms. This approach allows us to directly assess the differences arising from the underlying geometry representations and numerical methods. As a result, some implementations may deviate from recommended or highly optimized usage patterns for individual tools. However, all models were constructed using documented functionalities and standard features provided by each simulator.

We found that overall the simulations of both calcium diffusion (Figs 3–8 and Figs A–D in S1 Appendix) and calcium–calmodulin reaction–diffusion (Figs 9–14 and Figs E, G, and H in S1 Appendix) produced highly consistent results across the different simulation tools, despite their distinct underlying deterministic and stochastic algorithms. Previous studies provide a theoretical context for interpreting this agreement. At low molecular copy numbers, stochastic approaches capture fluctuations arising from the discrete nature of molecular interactions that are absent from deterministic concentration-based descriptions [16,28,55,71,72]. However, the consequences of these fluctuations for the simulated outcome are system dependent. Kummer et al. [73], for example, showed in models of calcium oscillations that the transition between stochastic and deterministic behavior depends not only on molecular copy number but also on the underlying system dynamics. Dobrzyński et al. [27] showed that under low-molecule-number and diffusion-limited conditions, spatial stochastic methods could predict the same average behavior while differing in the magnitude of the predicted fluctuations. Conversely, in nonlinear or excitable signaling systems, stochastic fluctuations can lead to qualitatively different dynamics. Denizot et al. [70] observed spontaneous calcium signals in stochastic models of fine astrocytic processes that were absent from the corresponding deterministic mean-field model, while Antunes and De Schutter [74] showed that stochasticity resulted in probabilistic induction of long-term depression in a Purkinje cell spine signaling network. In the calcium–calmodulin system considered here, although local molecular copy numbers can be small, the model does not contain the excitable or positive-feedback mechanisms that amplify fluctuations into distinct signaling events in these previous studies. We therefore expected stochasticity to manifest primarily as run-to-run variability rather than qualitatively different signaling regimes, while quantitative differences between deterministic and stochastic mean behavior could still occur. The overall agreement observed across the simulation tools is consistent with this expectation.

Since our 1D model did not include processes whose effects on the calcium dynamics depend on the surface area (such as calcium channels and exchangers), the differences in the surface-to-volume ratio between the cuboid and cylinder morphologies in the NeuroRD and NEURON simulators did not result in different calcium dynamics. However, if such processes are included, differences in the results of the two simulators could be expected if the channel densities per surface area were not scaled up or down in proportion. Some case-specific differences were observed, particularly for 3D diffusion in the cuboid morphology with NeuroRD (Fig 4) and in more complex astroglial morphologies with NEURON, such as the mouse Bergmann glial [33] (Fig 5) and human cortical astrocyte [32] (Fig 7) branchlet morphologies. In reaction–diffusion simulations, transient spatial heterogeneity in Ca_4_CaM distribution was observed at early time points. In particular, Ca_4_CaM formation was initially concentrated near the calcium injection site and, in some morphologies, appeared to remain localized in specific subregions separated by narrow branchlets. This behavior reflects diffusion-limited spread through geometrical constrictions, which can localize downstream reactions by temporarily restricting the spread of calcium ions [48,54]. Similar diffusion-limiting effects of narrow cellular structures have also been described in dendritic spines, where thin spine necks can transiently confine calcium and signaling molecules within individual synapses [75,76]. However, as the simulation progressed, Ca_4_CaM became more uniformly distributed throughout the morphology across all simulation tools, indicating that diffusion over longer timescales overcomes these local geometric constraints and bottlenecks (Fig 14). In the present study, calcium was introduced from one localized source, corresponding to a potential synaptic input site. More specifically, the injection region was defined as a finite volume rather than a single point, which helped to ensure consistent calcium initialization between the simulation tools. From the injection site, the ions were allowed to diffuse freely throughout the cytosolic volume. Future work could extend this framework by incorporating multiple calcium sources and intracellular compartments, such as the endoplasmic reticulum and cytosolic domains, to investigate how internal compartmentalization further shapes spatiotemporal signaling dynamics.

Although all four simulation tools are suitable for studying the calcium diffusion model and the reaction–diffusion calcium–calmodulin pathway model described in Tables 4 and 5, there are clear differences between the tools. In general, the usability of the tools for these specific use cases varied. In some tools, outdated documentation introduced some uncertainty for different simulations, for example, regarding the state-of-the-art possibilities for simulating different dimensions and geometries for diffusion and reaction–diffusion simulations. However, when it came to implementing the reactions and diffusion in each tool, the process was straightforward with the built-in reaction–diffusion components. For all of the tools, the determination of the reactants and the rate constants was intuitive and easy to modify.

Each of the selected simulation tools utilizes cell morphologies in different formats, which can be inconvenient to users. MCell uses polygonal surface meshes, STEPS volumetric meshes, NeuroRD cubic meshes, and NEURON skeletal morphologies. Several tools exist to modify morphologies between different formats (see, e.g., [42]). Of the selected simulation tools, only MCell and STEPS can utilize real 3D cell morphologies obtained from electron microscopy (EM) images converted, for example, into OBJ files. Especially for MCell, after reconstructing the EM images into a suitable format, their implementation was fairly user-friendly as well. In NeuroRD, the implementation of complex realistic morphologies requires—to our understanding—more experience on the practicalities of the tool and some manual work to connect, for example, each nanoprocess to a correct segment. A tool called ShapeShifter (https://github.com/neurord/ShapeShifter) is currently available to reduce the complexity of the morphology, which could be useful to create simpler geometric representations for NeuroRD. However, this tool has been tested currently only for morphologies in SWC or GENESIS [22] P file formats and with 3 point somata. For this reason, in this study realistic morphology simulations with NeuroRD were not performed. Converting surface-based morphologies into skeleton-based representations required by NEURON is not entirely straightforward. Existing approaches include VolRoverN, which generates reduced multicompartmental cable models from surface meshes and exports them in NEURON HOC format [77]. Other tools facilitate conversion between neuronal morphology formats and standardization into the SWC representation [78]. Nevertheless, representing highly complex astrocyte morphologies, such as Bergmann glial branchlets [33], within skeleton-based frameworks remains challenging, as astrocytes often contain dense branching patterns and loop-like structures that deviate from the tree-like assumptions underlying many morphology representations. Consequently, obtaining suitable SWC representations for such morphologies may require additional preprocessing or simplification. In the following, we describe the approach adopted here.

In this study, the original surface meshes were converted into SWC morphologies via volumetric voxelization followed by morphological closing and extraction of a connectivity-constrained voxel skeleton. This enabled their use within the NEURON 3D rxd framework. Although the resulting SWC representation is inherently tree-structured, the use of a dense voxel-derived skeleton ensures that spatially adjacent branches are represented by closely spaced segments. Upon discretization into a 3D finite-volume voxel grid in NEURON 3D rxd, these neighboring segments can become reconnected in space, allowing partial recovery of loop-like features present in the original morphology. This contrasts with classical 1D cable representations in NEURON, where such topological features cannot be preserved. To maintain geometric accuracy, morphology-specific diameter scaling was applied to match the voxelized volume to the original surface mesh, resulting in good agreement on global morphometric properties, including total volume and surface-to-volume ratio (Table 2). Despite these efforts, differences in geometric representation between NEURON and mesh-based simulators such as MCell and STEPS remain (Fig 2). In particular, the cubic voxel discretization in NEURON can distort fine structural features, such as narrow constrictions or thin processes, potentially reducing local diffusion barriers. Although increasing spatial resolution (i.e., reducing voxel size) would improve geometric accuracy, achievable resolution was limited by computational constraints, as finer discretizations substantially increase memory requirements in NEURON 3D rxd. Moreover, even at higher resolutions, voxel-based representations approximate curved surfaces less accurately than tetrahedral meshes, which can conform more closely to complex geometries. Comparison of the NEURON rxd and deterministic STEPS diffusion simulations with the corresponding stochastic simulations suggests that the observed discrepancies are more likely related to differences in geometrical representation than to the deterministic or stochastic solver formulation. For the deterministic reaction–diffusion simulations with STEPS, computational constraints required coarsening of the geometries. To enable a direct comparison of solver formulations, the corresponding stochastic STEPS simulations were repeated using the same coarsened geometries; these comparisons are presented in S1 Appendix. Nevertheless, diffusion profiles remained overall consistent across simulation platforms, indicating that the adopted conversion and discretization approach provides a reasonable compromise between geometric fidelity and computational feasibility.

Creating the cuboid and cylinder morphologies was intuitive and fast in most of the tools. With MCell and STEPS, simple geometries could be generated directly or imported with minimal preprocessing, making their setup user-friendly for diffusion simulations. Also, with NEURON, the creation of simple morphologies is clear, especially in 1D simulations with the neuron h submodule, and the documentation for it is up to date. As expected for NeuroRD, it required more effort to ensure a correct dimensionality of the geometry in 1D and 3D simulations. Understanding the implementation of the correct simulation dimensions was, in our experience, the most challenging practicality of the NeuroRD tool, especially to ensure the correct dimensionality of the diffusion dynamics. In general, simple geometries provide a convenient and reliable starting point for all tools, although the level of abstraction and user input required varies between implementations.

In addition to different cell morphology formats, simulation tools use different formats when dividing the volume into smaller subvolumes. Of the selected tools, STEPS uses tetrahedra and NeuroRD and NEURON 3D rxd cubic voxels. When running MCell through CellBlender, the morphology consists of one solid object. However, post-simulation, some manual manipulation can be introduced to create artificial tetrahedra (subvolumes) and bins to the morphology so that we can more clearly analyze different simulation tools in the diffusion simulations. The modeling of biological systems with complex geometries can be achieved with tetrahedra equally well or better than with cubes [17]. The quality of tetrahedra should be controlled; in particular, the lengths of the tetrahedral edges should not differ to such a degree that one tetrahedron is connected to more than four other tetrahedra [17]. In addition to quality control included in tetrahedral generators such as TetGen [79], Gmsh, and CUBIT, STEPS allows measuring the relationship between radius and edge, as well as the standard deviation for volume [17].

Implementing the correct inputs based on the available documentation for each tool was, in general, a clear task. However, evaluating whether to implement the input as the number of ions or the concentration required some thought to be able to adequately analyze the results from the different tools. For example, for NEURON rxd it was found to be more convenient to set the input as the concentration and later transform it back to the number of ions, whereas for MCell the most user-friendly way was to give the input as the number of ions in the CellBlender interface. In STEPS, input can be specified either as concentration or directly as molecule or ion count, as the solver operates on integer molecule and ion numbers within individual tetrahedral subvolumes rather than tracking individual particles. In this study, inputs in STEPS were implemented as a specified number of ions to remain consistent with those of the other simulation tools. Additionally, for the NeuroRD simulations, the input was given as a nanomolarity over a specific rate. Thus, the time course of injection of calcium ions was slightly different in NeuroRD compared to the other tools. Because the tools utilize different approaches for compartments, subvolumes, and other volume-related simulation details, it was considered a more robust choice to utilize the molecule count in this study.

The simulation wall-clock times varied between the tools. In the calcium–calmodulin reaction–diffusion benchmark considered here, STEPS exhibited longer runtimes compared to particle-based simulators such as MCell. However, this behavior should be interpreted in the context of the specific model and solver formulation. Previous work by Hepburn et al. [17] has demonstrated that STEPS can achieve high computational efficiency in spatial stochastic simulations, particularly in systems with localized reactions, membrane-associated processes, or lower effective reaction event densities. In MCell, running one simulation seed with the reaction–diffusion model took approximately 10 minutes with a time step of 10^-5^ s, regardless of the simulated morphology, while in STEPS it took hours due to the large number of subvolumes. The calcium–calmodulin system considered here involves high molecule counts, frequent diffusion events, and exclusively cytosolic bimolecular reactions, without membrane-localized reactions or rare stochastic events. In such cases, the event-based, mesh-resolved formulation used by STEPS incurs substantial overhead due to diffusion bookkeeping across large numbers of tetrahedra. By contrast, particle-based methods benefit from continuous-space diffusion and collision-limited reactions, avoiding volumetric mesh traversal. Additionally, the use of uniform volumetric discretization in the present study may further contribute to the computational cost observed in STEPS. Employing spatially adaptive discretization strategies, such as finer subvolumes in narrow processes and coarser subvolumes in larger cytosolic regions, could improve computational efficiency while preserving geometric fidelity. However, in the present study, a uniform discretization was deliberately adopted to ensure consistency across simulation tools. We therefore interpret the observed performance differences as model-dependent and anticipate that STEPS would be more efficient in scenarios involving sparse reactions, strong compartmentalization, or channel-mediated calcium fluxes, such as endoplasmic reticulum-mediated calcium-induced calcium release dynamics. In NEURON rxd, simulating 1D diffusion and reaction–diffusion models was computationally rather efficient. However, the computational load increased significantly with the number of subvolumes. As an example, the simulation wall-clock time with NEURON ranged from 0.17 s (1D diffusion) to hours (3D reaction–diffusion simulation from the simplified geometry to Bergmann glial branchlet morphology 2) with our resources. However, the simulation wall-clock time with NeuroRD ranged from 0.014 s (1D diffusion) to around 2 min (3D reaction–diffusion simulation). It is important to note that for NeuroRD 3D simulations, a significantly smaller number of subvolumes was used. However, validation of the tool has presented promising computational efficiency [18].

Beyond the tools considered here, there are other free open-source simulation approaches that address complementary modeling scales, such as Smoldyn [14], MesoRD [19], Virtual Cell [24,25], GENESIS/Chemesis [22,23], MOOSE [26], ASTRO [21], DuneCopasi [80], and SMART [81]. Recent particle-based approaches, such as PyRID [82], enable simulations of spatially resolved molecular assemblies with explicit steric interactions and crowding, complementing continuum reaction–diffusion solvers. At the network level, a large-scale simulation framework in NEST [83] has been developed that couples astrocytic dynamics with neuronal activity in spiking neural networks [84]. Other neural network simulators include ARACHNE [85] and Brian2 [86,87]. In addition to open-source simulation tools mentioned in this study, commercial software platforms are available for modeling biological systems in different scales. Sensitivity analysis or parameter estimation is also highly important in reaction–diffusion modeling. For example, Uncertainpy [88] provides built-in support for NEURON and NEST models, and physics-informed neural networks (PINNs) [89] could also prove useful. Although these approaches were beyond the scope of the present study, future research will benefit from a systematic analysis of how detailed intracellular reaction–diffusion solvers can be combined with network-level models to bridge molecular, cellular, and network scales.

This study analyzed the usability, geometry handling, implementation effort, flexibility, and cross-simulator consistency of diffusion and reaction–diffusion schemes in four widely used simulation tools—MCell, STEPS, NeuroRD, and the NEURON rxd module. In addition to their typical application to neuronal morphologies, we examined how these tools represent complex subcellular geometries of astroglial branchlets. Our analysis highlights both the strengths and limitations of each platform, providing insights into their suitability for integrating reaction–diffusion processes with future network-level simulations in co-simulation frameworks. We observed a high degree of consistency between the tools for both simplified geometries and realistic astroglial branchlet morphologies. For the latter, partial astrocyte geometries reconstructed from EM data [31–33] enabled a systematic assessment of how morphological complexity influences simulation outcomes across platforms. Our findings emphasize the effect of morphology for spatially and temporally dependent calcium diffusion and calcium–calmodulin reaction–diffusion systems. Further studies, with more complex biochemical models, can dive deeper with more detailed insights for the role of the morphology. Taken together, these results clarify the capabilities and limitations of current tools for reaction–diffusion modeling in complex cellular geometries and provide guidance for their use in future studies.

## Supporting information

S1 Appendix Additional data to analyze simulation tools and morphologies. (PDF)
