# Supplementary material for: Modeling reaction–diffusion processes in simplified to complex astroglial branchlet geometries using different simulation tools
Source: PLoS Comput Biol. 2026 Oct 28;22(10):e1014878. doi: 10.1371/journal.pcbi.1014878 (PMC13716318; doi:10.1371/journal.pcbi.1014878)
Supplement: Supplementary file 1 [file pcbi.1014878.s001.pdf]

S1 Appendix

Additional data to analyze simulation tools and morphologies

Laura Keto◑, Saana Seppälä◑, Derek Ndubuaku, Annika Mäki, Tuomo Mäki-Marttunen, Marja-Leena Linne, Tiina Manninen*

Faculty of Medicine and Health Technology, Tampere University, Tampere, Finland

◑These authors contributed equally to this work.
* tiina.manninen@tuni.fi

This appendix presents additional data for the article titled: Modeling reaction–diffusion processes in simplified to complex astroglial branchlet geometries using different simulation tools.

3D calcium diffusion in real cell morphologies

Fig A presents 3D calcium diffusion results with three suitable tools, MCell (`https://mcell.org`) [1–3], STEPS (`https://steps.sourceforge.net`) [4], and NEURON (`https://www.neuronsimulator.org/`) [5, 6], using the mouse Bergmann glial branchlet morphology 1 [7] (Fig 1C in the article). Calcium ions were injected into the morphology from one possible synapse location. We plotted the results with 50 distance bins created with all the tools. For MCell and STEPS, we show the mean and standard deviation of 20 runs, in addition to a single realization of stochastic runs and deterministic STEPS result. As can be seen in Fig A, the mean and standard deviation of MCell and STEPS results are consistent, deterministic STEPS follows closely the mean values, and single realizations of stochastic runs from MCell and STEPS mostly land inside the standard deviation. However, NEURON shows some differences compared to the other simulation results. When looking at the root mean square deviation (RMSD), normalized root mean square deviation (NRMSD), and Pearson Correlation Coefficient for these simulation results, we can see that the comparison of the mean results of MCell and STEPS as well as the stochastic and deterministic STEPS results are more similar than when comparing them to NEURON (Tables A–C). Tables A and C also show values for Figs 5–7 presented in the article.

Similarly to Fig A, Fig B presents 3D calcium diffusion results with three suitable tools, MCell, STEPS, and NEURON, using mouse cortical astrocyte branchlet morphology 2 [8] (Fig 1F in the article). Compared to Fig A, the MCell, STEPS, and NEURON results are more consistent throughout the simulation in Fig B. Between Figs A and B, the RMSD, NRMSD, and Pearson Correlation Coefficient values are similar when comparing the mean results of MCell and STEPS as well as the stochastic and deterministic STEPS results (Tables A–C).

Fig C presents 3D calcium diffusion results with three suitable tools, MCell, STEPS, and NEURON, using human cortical astrocyte branchlet morphology 2 [9] (Fig 1H in the article). Similarly to Figs A and B, the mean and standard deviation of MCell and STEPS results are consistent in Fig C. Of these three figures (Figs A–C), Fig C shows the most similar results between tools as can be confirmed in Tables A–C.

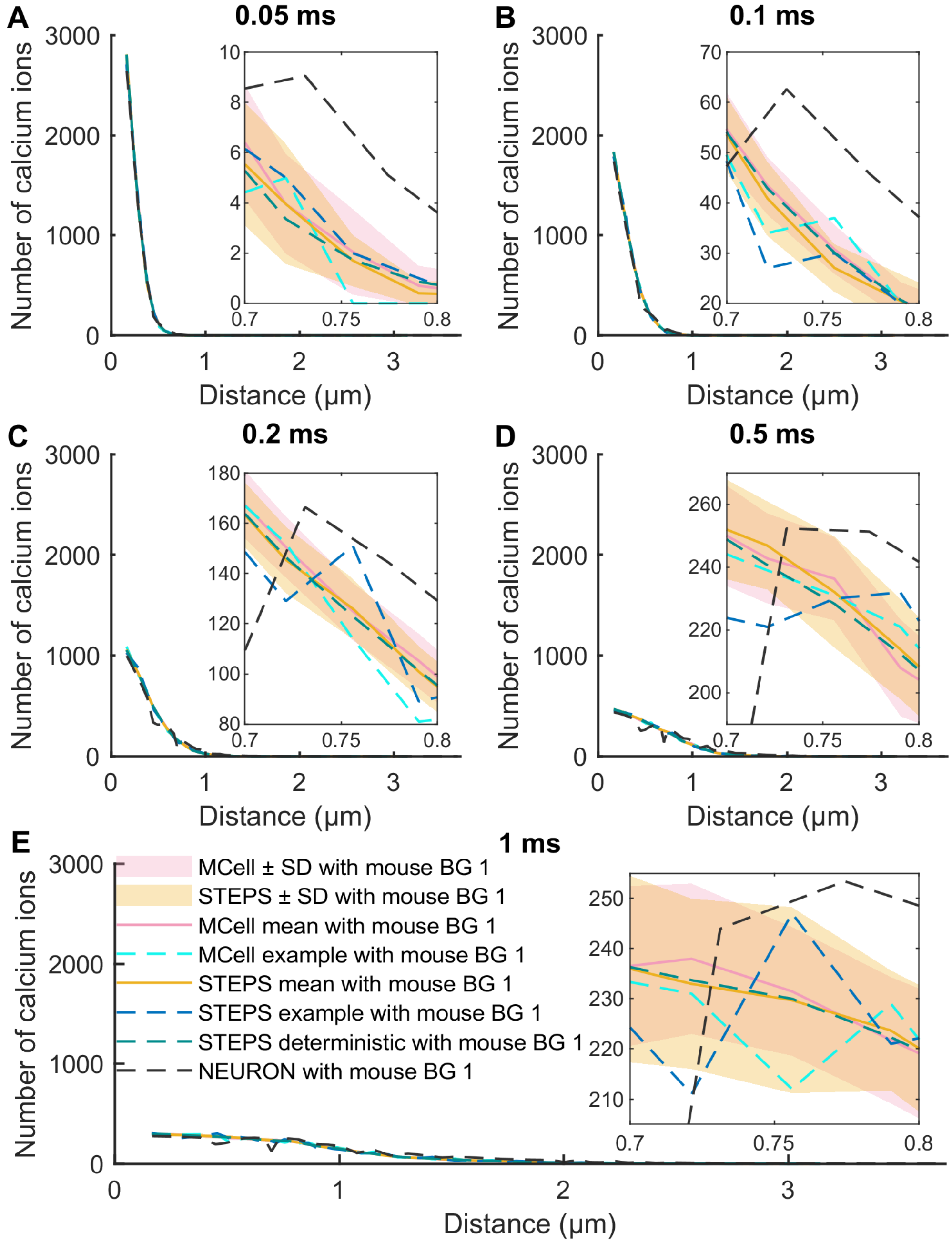

Fig A. 3D calcium diffusion simulations in the mouse Bergmann glial branchlet morphology 1 (Mouse BG 1) show similar results with MCell, STEPS, and NEURON `rxd`. Mean and standard deviation of 20 simulation runs shown with MCell and STEPS together with single realizations of MCell and STEPS runs and deterministic STEPS and NEURON simulations. Number of calcium ions plotted as a function of geodesic distance from the injection point at time points of (A) 0.05 ms, (B) 0.1 ms, (C) 0.2 ms, (D) 0.5 ms, and (E) 1 ms with insets. Shaded area indicates ±SD.

Fig D shows the morphological representation of calcium diffusion in MCell, STEPS,

Table A. Root mean square deviation (RMSD) calculated when comparing calcium diffusion simulation results between two simulation tools for astrocyte branchlet morphologies. Different time points 0.05 ms, 0.1 ms, 0.2 ms, 0.5 ms, and 1 ms represent subfigures A–E, respectively, plotted from calcium diffusion simulations in Figs 5–7 in the article and Figs A–C in this appendix. STEPS det and NEURON are deterministic simulations, others are stochastic simulations of which we used the mean of 20 simulations in the calculations.

Time point	Tools	Fig A: Mouse BG 1	Fig 5: Mouse BG 2	Fig 6: Mouse CX 1	Fig B: Mouse CX 2	Fig 7: Human CX 1	Fig C: Human CX 2
**0.05 ms**	**MCell & STEPS**	13.3	0.213	3.19	11.1	5.65	0.780
	**MCell & NEURON**	18.7	0.457	66.6	65.8	28.6	9.10
	**STEPS & NEURON**	26.7	0.249	66.2	75.5	23.1	9.88
	**STEPS & STEPS det**	1.19	0.0217	1.59	0.97	1.04	0.237
	**STEPS det & NEURON**	26.3	0.262	66.9	74.8	24.1	10.1
**0.1 ms**	**MCell & STEPS**	6.17	1.24	3.68	7.94	2.19	4.17
	**MCell & NEURON**	26.1	3.04	63.9	47.5	30.4	19.9
	**STEPS & NEURON**	28.0	1.84	60.9	53.3	28.3	24.0
	**STEPS & STEPS det**	1.52	0.805	2.00	1.98	1.31	1.69
	**STEPS det & NEURON**	28.1	2.60	61.6	53.5	29.6	22.3
**0.2 ms**	**MCell & STEPS**	5.80	0.577	2.14	6.74	1.88	1.71
	**MCell & NEURON**	30.5	4.75	51.5	34.5	29.7	28.3
	**STEPS & NEURON**	32.1	4.55	50.8	39.8	30.1	30.0
	**STEPS & STEPS det**	2.06	1.04	2.27	2.34	1.75	1.51
	**STEPS det & NEURON**	32.1	3.78	49.3	38.2	30.7	28.5
**0.5 ms**	**MCell & STEPS**	3.45	2.93	2.21	3.51	1.51	4.60
	**MCell & NEURON**	28.0	7.12	31.2	25.6	25.2	30.0
	**STEPS & NEURON**	29.5	6.05	29.9	26.3	24.8	33.4
	**STEPS & STEPS det**	2.01	1.62	1.95	2.50	1.25	1.09
	**STEPS det & NEURON**	28.8	6.47	28.4	25.9	24.8	32.6
**1 ms**	**MCell & STEPS**	2.40	2.23	2.20	3.08	2.13	2.46
	**MCell & NEURON**	25.2	11.9	21.2	20.2	22.5	29.3
	**STEPS & NEURON**	24.9	12.7	21.7	20.2	22.5	30.2
	**STEPS & STEPS det**	1.10	1.64	1.67	2.02	1.90	1.92
	**STEPS det & NEURON**	24.6	12.4	21.7	20.1	21.0	30.5

and NEURON for Bergmann glial branchlet morphology 1, mouse cortical astrocyte branchlet morphology 2, and human cortical astrocyte branchlet morphology 2. The spatial distribution patterns of calcium ions are visually similar across the three simulators; however, there are clear differences between the morphologies. Clearly, calcium diffusion is constrained by the underlying branchlet geometry. In Bergmann glial branchlet morphology 1, calcium ions are more spread than in the two cortical astrocyte branchlet morphologies. These results are consistent with the quantitative trends observed in Figs A–C.

Table B. Normalized root mean square deviation (NRMSD) calculated by dividing the RMSD by the range of values when comparing calcium diffusion simulation results between two simulation tools for astrocyte branchlet morphologies. Different time points 0.05 ms, 0.1 ms, 0.2 ms, 0.5 ms, and 1 ms represent subfigures A–E, respectively, plotted from calcium diffusion simulations in Figs A–C in this appendix. STEPS det and NEURON are deterministic simulations, others are stochastic simulations of which we used the mean of 20 simulations in the calculations. The NRMSD values are always below 9%.

**Time point**	**Tools**	**Fig A: Mouse BG 1**	**Fig B: Mouse CX 2**	**Fig C: Human CX 2**
**0.05 ms**	**MCell & STEPS**	0.49	0.44	0.016
	**MCell & NEURON**	0.69	2.6	0.19
	**STEPS & NEURON**	0.95	2.9	0.21
	**STEPS & STEPS det**	0.042	0.037	0.0050
	**STEPS det & NEURON**	0.94	2.9	0.21
**0.1 ms**	**MCell & STEPS**	0.34	0.53	0.099
	**MCell & NEURON**	1.5	3.2	0.47
	**STEPS & NEURON**	1.5	3.4	0.57
	**STEPS & STEPS det**	0.083	0.13	0.040
	**STEPS det & NEURON**	1.5	3.4	0.53
**0.2 ms**	**MCell & STEPS**	0.57	0.72	0.049
	**MCell & NEURON**	3.0	3.7	0.82
	**STEPS & NEURON**	3.0	4.1	0.86
	**STEPS & STEPS det**	0.19	0.24	0.043
	**STEPS det & NEURON**	3.0	4.0	0.82
**0.5 ms**	**MCell & STEPS**	0.75	0.58	0.18
	**MCell & NEURON**	6.1	4.2	1.2
	**STEPS & NEURON**	6.4	4.3	1.3
	**STEPS & STEPS det**	0.43	0.41	0.043
	**STEPS det & NEURON**	6.2	4.3	1.3
**1 ms**	**MCell & STEPS**	0.79	0.68	0.12
	**MCell & NEURON**	8.3	4.4	1.4
	**STEPS & NEURON**	8.4	4.4	1.5
	**STEPS & STEPS det**	0.37	0.45	0.093
	**STEPS det & NEURON**	8.3	4.4	1.5

Table C. Pearson Correlation Coefficient calculated when comparing calcium diffusion simulation results between two simulation tools for astrocyte branchlet morphologies. Different time points 0.05 ms, 0.1 ms, 0.2 ms, 0.5 ms, and 1 ms represent subfigures A–E, respectively, plotted from calcium diffusion simulations in Figs 5–7 in the article and Figs A–C in this appendix. STEPS det and NEURON are deterministic simulations, others are stochastic simulations of which we used the mean of 20 simulations in the calculations.

Time point	Tools	Fig A: Mouse BG 1	Fig 5: Mouse BG 2	Fig 6: Mouse CX 1	Fig B: Mouse CX 2	Fig 7: Human CX 1	Fig C: Human CX 2
**0.05 ms**	**MCell & STEPS**	1.000	1.000	1.000	1.000	1.000	1.000
	**MCell & NEURON**	1.000	1.000	0.990	0.988	0.999	1.000
	**STEPS & NEURON**	0.999	1.000	0.990	0.985	0.999	1.000
	**STEPS & STEPS det**	1.000	1.000	1.000	1.000	1.000	1.000
	**STEPS det & NEURON**	0.999	1.000	0.990	0.986	0.999	1.000
**0.1 ms**	**MCell & STEPS**	1.000	1.000	1.000	1.000	1.000	1.000
	**MCell & NEURON**	0.997	1.000	0.985	0.987	0.998	1.000
	**STEPS & NEURON**	0.998	1.000	0.986	0.985	0.998	1.000
	**STEPS & STEPS det**	1.000	1.000	1.000	1.000	1.000	1.000
	**STEPS det & NEURON**	0.998	1.000	0.986	0.985	0.998	1.000
**0.2 ms**	**MCell & STEPS**	1.000	1.000	1.000	1.000	1.000	1.000
	**MCell & NEURON**	0.991	1.000	0.985	0.989	0.997	0.999
	**STEPS & NEURON**	0.991	1.000	0.985	0.986	0.996	0.999
	**STEPS & STEPS det**	1.000	1.000	1.000	1.000	1.000	1.000
	**STEPS det & NEURON**	0.991	1.000	0.986	0.987	0.996	0.999
**0.5 ms**	**MCell & STEPS**	1.000	1.000	1.000	1.000	1.000	1.000
	**MCell & NEURON**	0.978	1.000	0.991	0.990	0.996	0.998
	**STEPS & NEURON**	0.977	1.000	0.992	0.990	0.996	0.998
	**STEPS & STEPS det**	1.000	1.000	1.000	1.000	1.000	1.000
	**STEPS det & NEURON**	0.978	1.000	0.993	0.990	0.996	0.998
**1 ms**	**MCell & STEPS**	1.000	1.000	1.000	1.000	1.000	1.000
	**MCell & NEURON**	0.970	1.000	0.994	0.992	0.996	0.998
	**STEPS & NEURON**	0.971	1.000	0.993	0.992	0.996	0.998
	**STEPS & STEPS det**	1.000	1.000	1.000	1.000	1.000	1.000
	**STEPS det & NEURON**	0.971	1.000	0.993	0.992	0.996	0.998

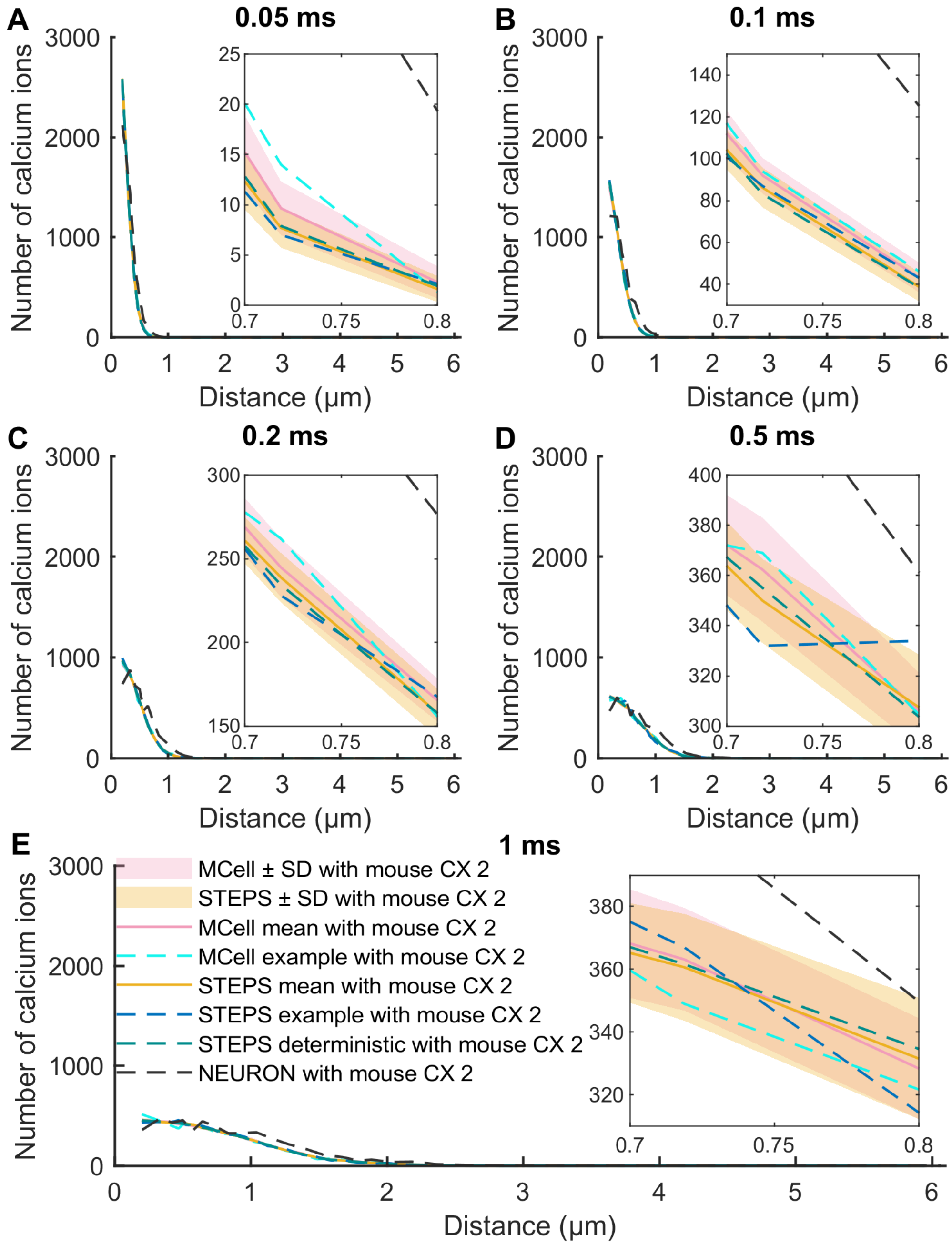

Fig B. 3D calcium diffusion simulations in the mouse cortical astrocyte branchlet morphology 2 (Mouse CX 2) show similar results with MCell, STEPS, and NEURON `rxd`. Mean and standard deviation of 20 simulation runs shown with MCell and STEPS together with single realizations of MCell and STEPS runs and deterministic STEPS and NEURON simulations. Number of calcium ions plotted as a function of geodesic distance from the injection point at time points of (A) 0.05 ms, (B) 0.1 ms, (C) 0.2 ms, (D) 0.5 ms, and (E) 1 ms with an inset. Shaded area indicates ±SD.

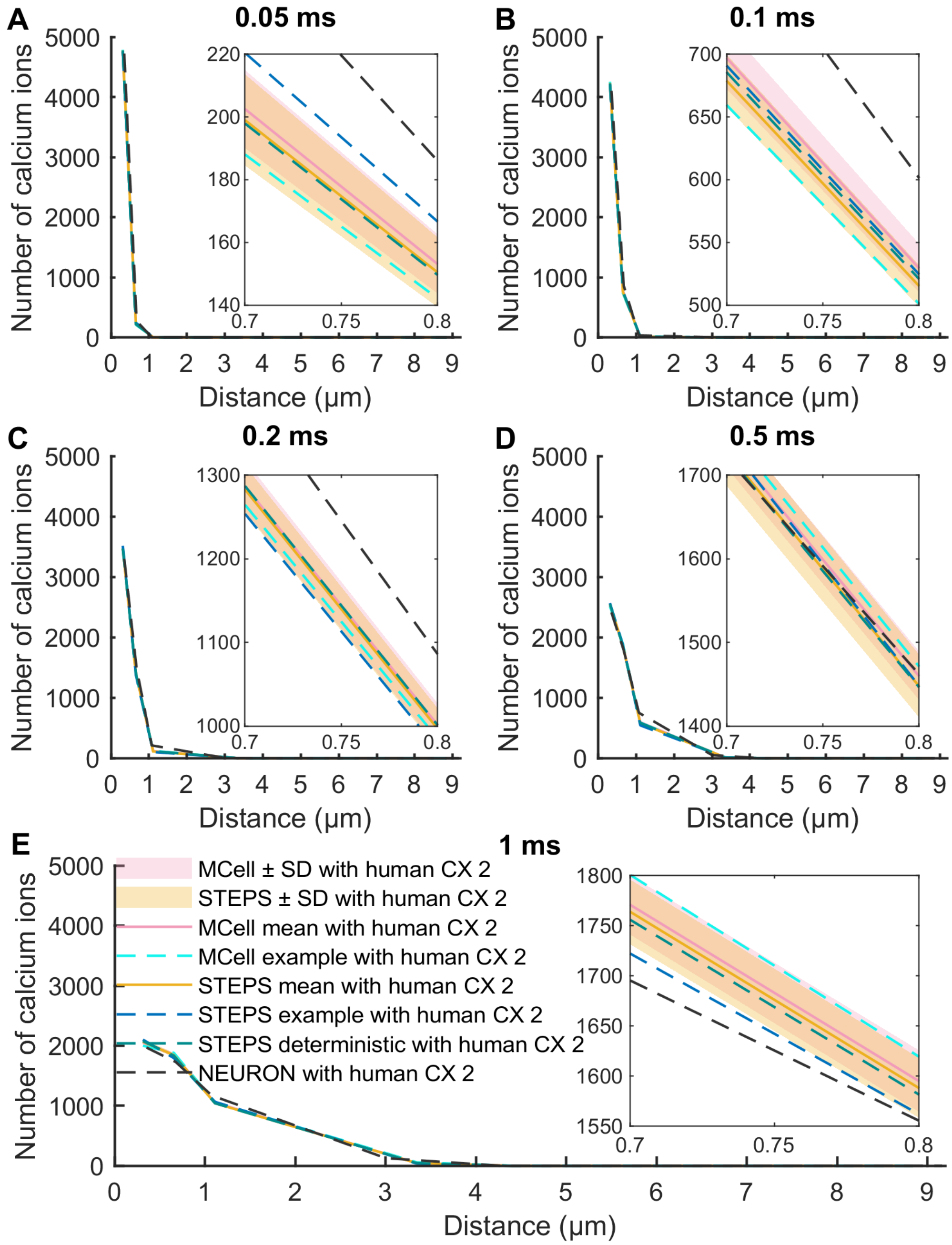

Fig C. 3D calcium diffusion simulations in the human cortical astrocyte branchlet morphology 2 (Human CX 2) show similar results with MCell, STEPS, and NEURON `rxd`. Mean and standard deviation of 20 simulation runs shown with MCell and STEPS together with single realizations of MCell and STEPS runs and deterministic STEPS and NEURON simulations. Number of calcium ions plotted as a function of geodesic distance from the injection point at time points of (A) 0.05 ms, (B) 0.1 ms, (C) 0.2 ms, (D) 0.5 ms, and (E) 1 ms with an inset. Shaded area indicates ±SD.

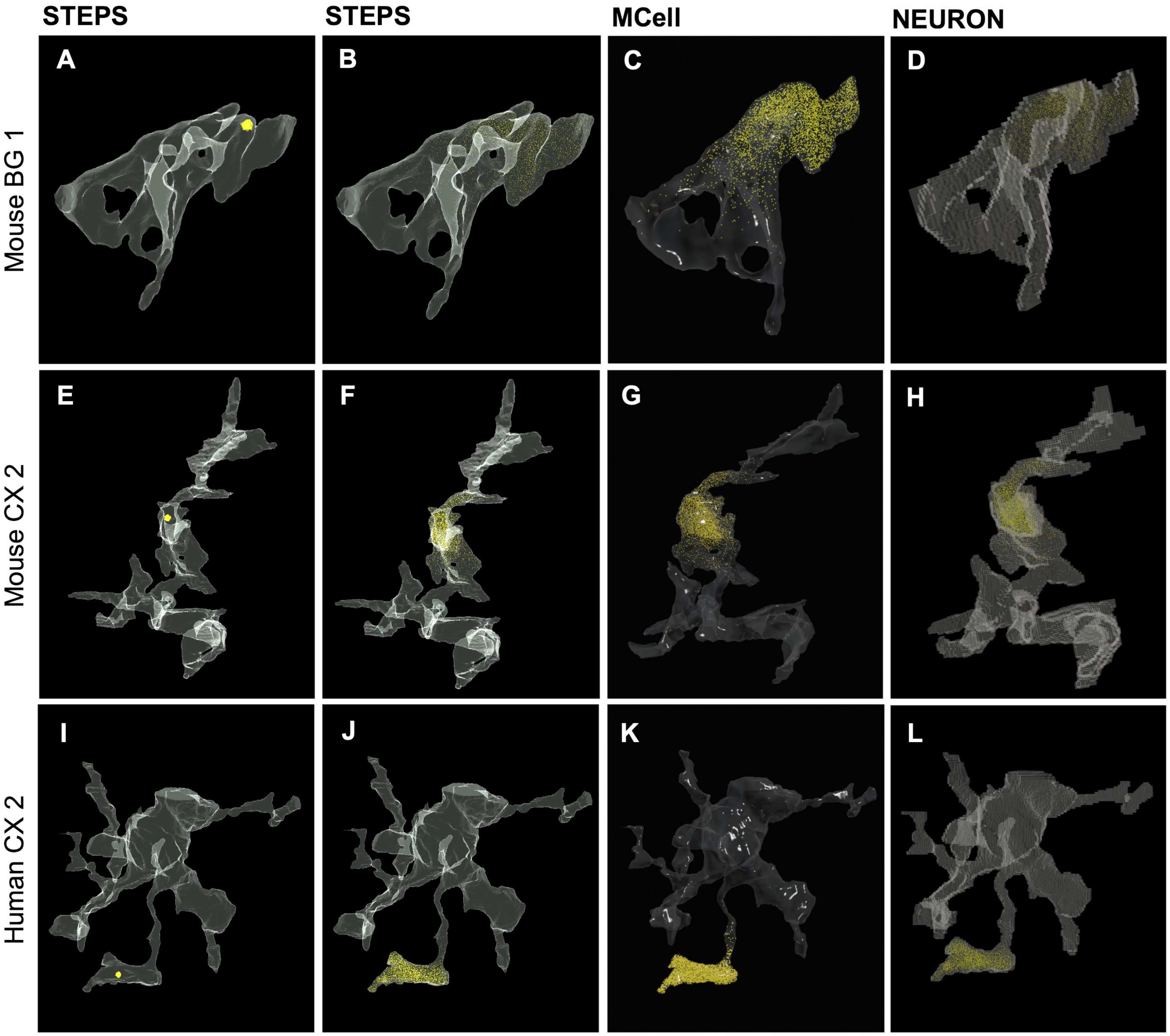

Fig D. Morphological presentation of the calcium diffusion simulations in astrocyte branchlet morphologies shows similar results with MCell, STEPS, and NEURON. (A, E, I) The initial time point in the beginning of the STEPS simulations for the branchlets of the mouse Bergmann glial morphology 1 (Mouse BG 1) [7], mouse cortical astrocyte morphology 2 (Mouse CX 2) [8], and human cortical astrocyte morphology 2 (Human CX 2) [9]. (B, F, J) Stochastic STEPS simulations at 1 ms. (C, G, K) MCell simulations at 1 ms. (D, H, L) NEURON simulations at 1 ms. Calcium ions are illustrated in yellow. For visualization of the NEURON simulations in this figure, voxels were rendered as uniform cuboids of edge length `dx` centered at voxel nodes to improve visual clarity.

Reaction–diffusion simulations with 3D diffusion in real cell morphologies

In Fig E, the morphological representations of the reaction–diffusion simulations are shown with MCell, STEPS, and NEURON for the same morphologies as in Fig D; Bergmann glial branchlet morphology 1, mouse cortical astrocyte branchlet morphology 2, and human cortical astrocyte branchlet morphology 2. The quantitative reaction–diffusion results for these morphologies are presented in Figs 11–13 in the article. Fig E shows a consistent reaction–diffusion behavior for calcium, calmodulin, and Ca_4CaM despite the different geometric representations used by the tools. Interestingly, for human cortical astrocyte branchlet morphology 2, a smaller number of Ca_4CaM molecules is formed with all the tools compared to other morphologies.

Tables D and E show the RMSD and Pearson Correlation Coefficient values for the mouse Bergmann glial branchlet morphology 2 [7] (Fig 1D in the article), mouse cortical astrocyte branchlet morphology 1 [8] (Fig 1E in the article), and human cortical astrocyte branchlet morphology 1 [9] (Fig 1G in the article) presented in Figs 11–13 in the article. While NRMSD was used as the primary measure in the article (Table 7), these additional measures are provided for completing the comparison between simulations. All values for Pearson Correlation Coefficients are ≥ 0.926, indicating similar trend within the compared simulations.

Table D. Root mean square deviation (RMSD) calculated when comparing reaction–diffusion simulation results between two simulation tools for astrocyte branchlet morphologies. Calcium, calmodulin, and Ca_4CaM present the model variables obtained from calcium–calmodulin reaction–diffusion simulations in Figs 11–13 in the article. NEURON uses deterministic simulations, others are stochastic simulations of which we used the mean of 20 simulations in the calculations.

Model variable	Tools	Fig 11: Mouse BG 2	Fig 12: Mouse CX 1	Fig 13: Human CX 1
	**MCell & STEPS**	12.4	10.0	12.1
	**MCell & NEURON**	14.8	166	30.6
**Calcium**	**STEPS & NEURON**	14.2	167	27.3
	**MCell & STEPS**	7.77	6.05	6.58
	**MCell & NEURON**	8.58	73.7	11.5
**Calmodulin**	**STEPS & NEURON**	8.82	74.7	12.4
	**MCell & STEPS**	0.853	1.97	1.97
	**MCell & NEURON**	0.644	5.98	3.94
**Ca_4CaM**	**STEPS & NEURON**	0.743	5.61	3.26

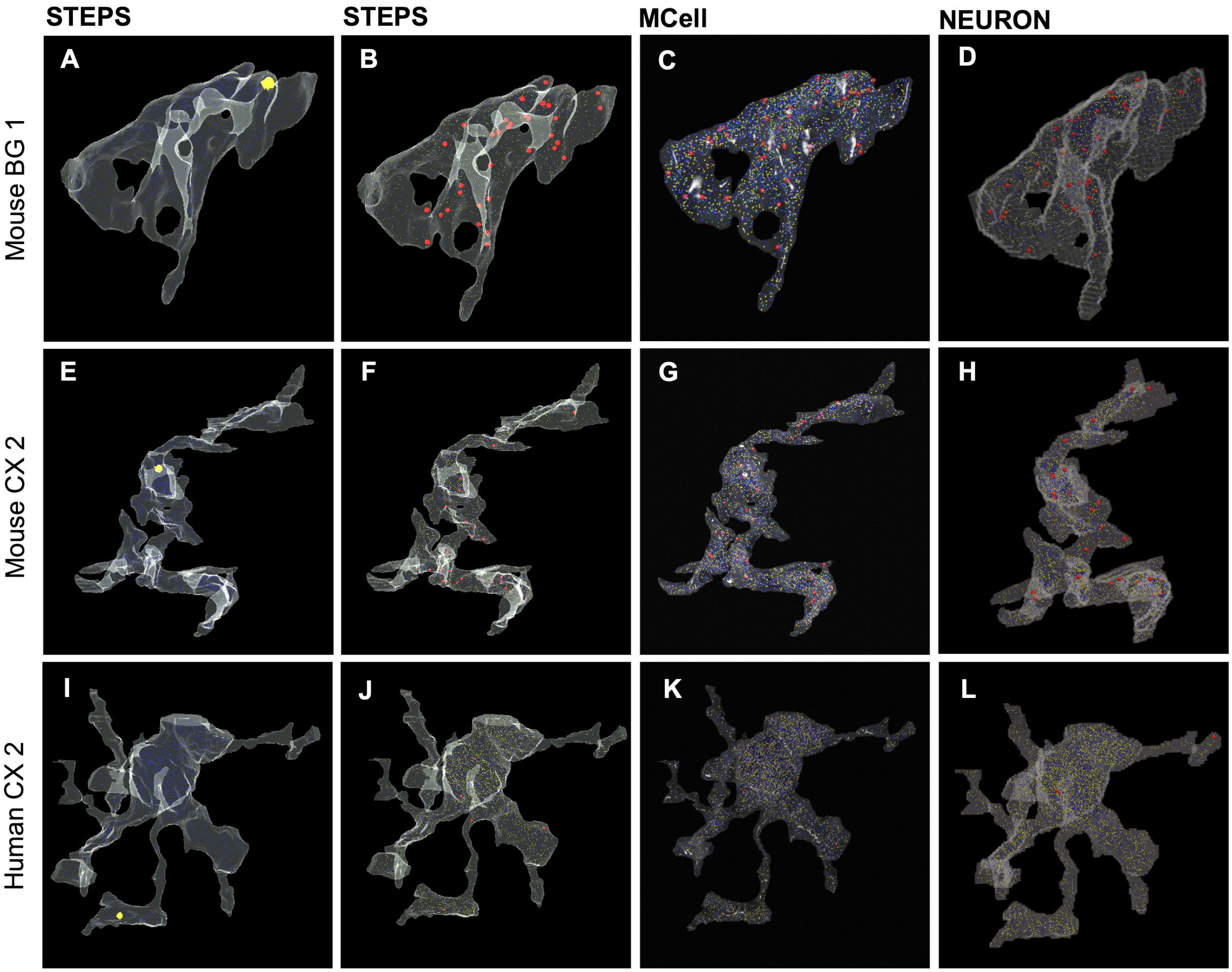

Fig E. Morphological presentation of the reaction–diffusion simulations in astrocyte branchlet morphologies shows similar results with MCell, STEPS, and NEURON. (A, E, I) The initial time point in the beginning of the STEPS simulations for the branchlets of the mouse Bergmann glial morphology 1 (Mouse BG 1) [7], mouse cortical astrocyte morphology 2 (Mouse CX 2) [8], and human cortical astrocyte morphology 2 (Human CX 2) [9]. (B, F, J) Stochastic STEPS simulations at 1 s. (C, G, K) MCell simulations at 1 s. (D, H, L) NEURON simulations at 1 s. Calcium ions are illustrated in yellow, calmodulin in blue, and Ca_4CaM in red. For visualization of the NEURON simulations in this figure, voxels were rendered as uniform cuboids of edge length `dx` centered at voxel nodes to improve visual clarity.

Table E. Pearson Correlation Coefficient calculated when comparing reaction–diffusion simulation results between two simulation tools for astrocyte branchlet morphologies. Calcium, calmodulin, and Ca_4CaM present the model variables obtained from calcium–calmodulin reaction–diffusion simulations in Figs 11–13 in the article. NEURON uses deterministic simulations, others are stochastic simulations of which we used the mean of 20 simulations in the calculations.

Model variable	Tools	Fig 11: Mouse BG 2	Fig 12: Mouse CX 1	Fig 13: Human CX 1
Calcium	MCell & STEPS	0.998	0.999	0.999
	MCell & NEURON	0.999	0.998	0.999
	STEPS & NEURON	0.999	0.998	0.999
Calmodulin	MCell & STEPS	0.998	0.999	0.999
	MCell & NEURON	0.999	0.999	0.999
	STEPS & NEURON	0.999	0.999	0.999
Ca_4CaM	MCell & STEPS	0.967	0.926	0.997
	MCell & NEURON	0.985	0.947	0.998
	STEPS & NEURON	0.982	0.950	0.998

Deterministic and stochastic reaction–diffusion simulations with 3D diffusion in simplified morphologies with STEPS

To enable a direct comparison of deterministic and stochastic reaction–diffusion simulations in spatial geometries, coarser tetrahedral meshes were generated because simulations using the original finely tetrahedralized meshes were computationally prohibitive with the deterministic STEPS TetODE solver. For the cylinder geometry, the number of tetrahedra was reduced from 164,139 to 8,907, while the mesh volume changed only from 1.571 to 1.518 μm^3 (3.4% decrease). For mouse cortical astrocyte branchlet morphology 1 [8], the number of tetrahedra was reduced from 73,839 to 7,162, with the mesh volume decreasing from 0.714 to 0.553 μm^3 (22.5% decrease). Fig F shows the original and coarsened representations of the cylinder and branchlet morphologies. The coarsened mesh preserves the overall branchlet geometry, although some of the finer morphological features are no longer resolved.

The same calcium–calmodulin reaction–diffusion model, initial molecule numbers, diffusion coefficients, and calcium injection protocol were used for the deterministic TetODE and stochastic Tetexact simulations. The stochastic results represent the mean of 20 Tetexact simulations and a single realization. The resulting calcium, calmodulin, and Ca_4CaM dynamics showed close agreement between the deterministic and stochastic approaches (Figs G and H). The NRMSD values for the branchlet morphology were 0.16% for calcium, 0.19% for calmodulin, and 1.6% for Ca_4CaM, with corresponding Pearson correlation coefficients of 1.000, 0.999, and 0.977, respectively. The RMSD values were 6.45, 4.31, and 1.51 for calcium, calmodulin, and Ca_4CaM, respectively.

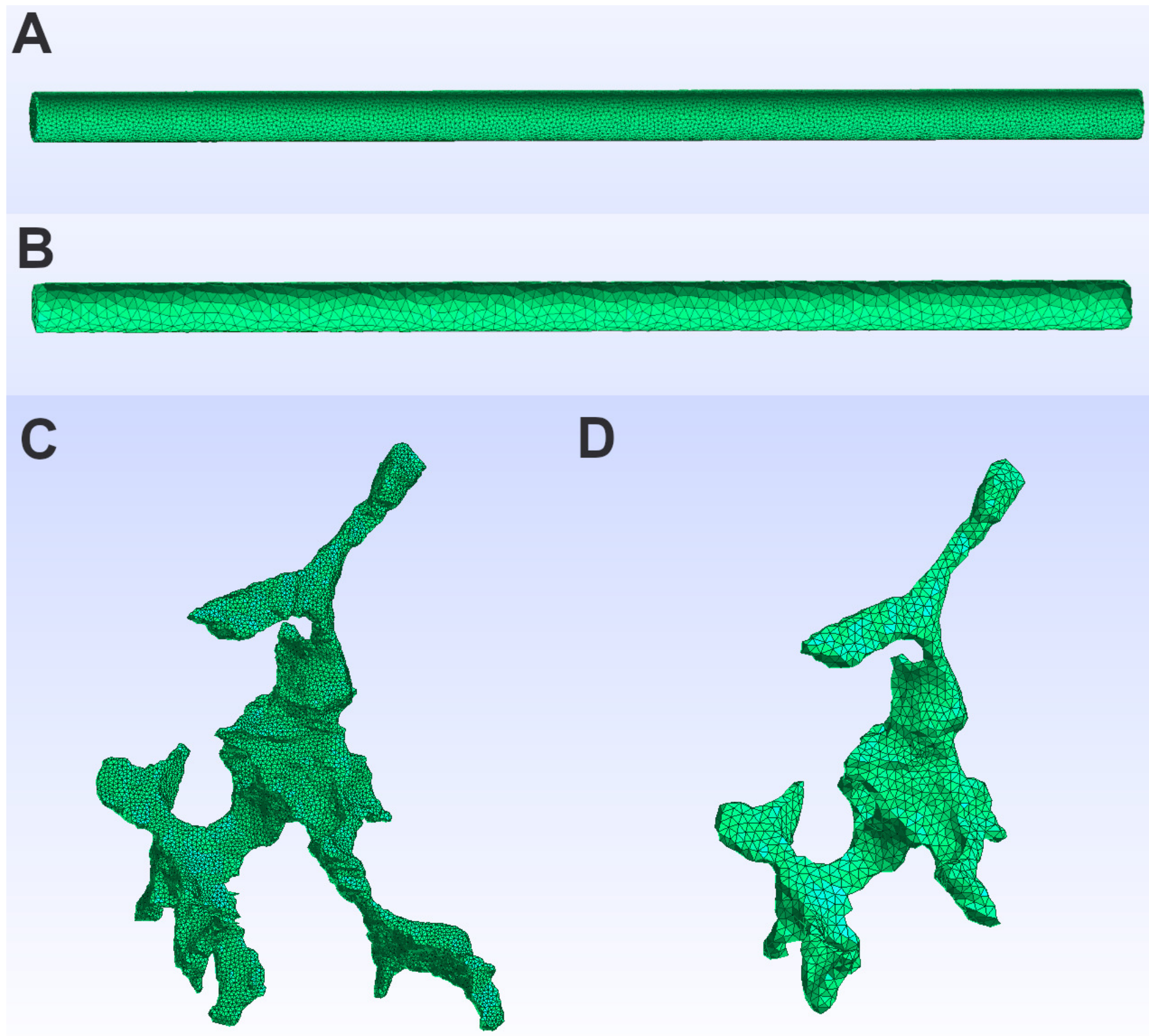

Fig F. Original and simplified cylinder and mouse cortical astrocyte branchlet morphologies used in deterministic and stochastic simulations with STEPS. (A) The original cylinder morphology used in the simulations in the article. (B) The simplified cylinder morphology used in deterministic and stochastic reaction–diffusion simulations with STEPS shown in Fig G. (C) The original mouse cortical astrocyte branchlet morphology 1 [8] used in most of the simulations in the article and in this appendix. (D) The simplified mouse cortical astrocyte branchlet morphology 1 [8] used in deterministic and stochastic reaction–diffusion simulations with STEPS shown in Fig H. Visualization was performed in Gmsh [10].

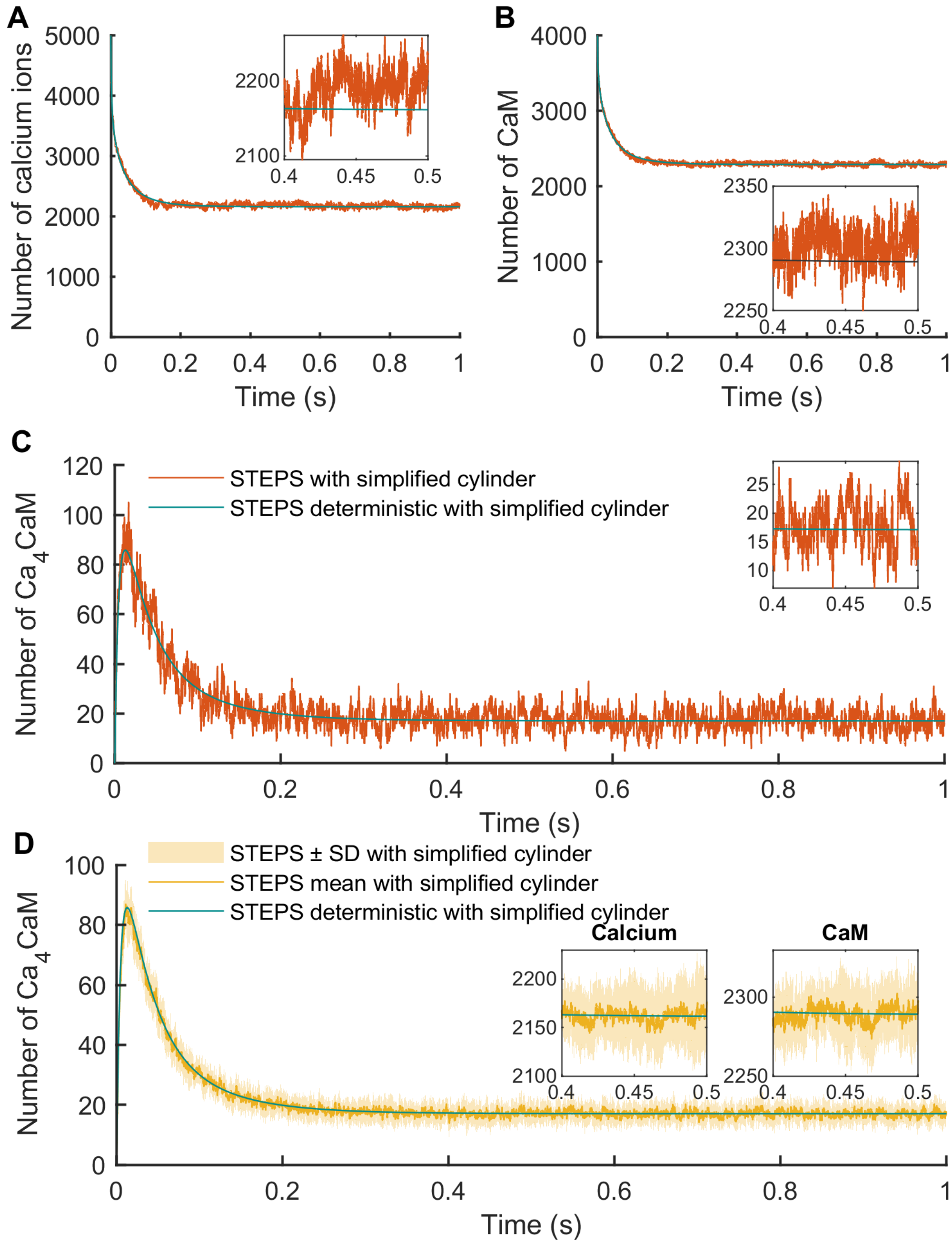

Fig G. Reaction–diffusion simulations with 3D diffusion in the simplified cylinder morphology show consistent results with deterministic and stochastic STEPS. Number of (A) calcium ions, (B) calmodulin (CaM) molecules, and (C) Ca_4CaM complexes plotted as a function of time from a single realization of a stochastic run with STEPS together with deterministic STEPS simulation, and (D) mean and standard deviation (shaded area indicates $\pm$SD) of Ca_4CaM from 20 STEPS simulation runs together with deterministic STEPS simulation plotted as a function of time with insets for calcium and calmodulin.

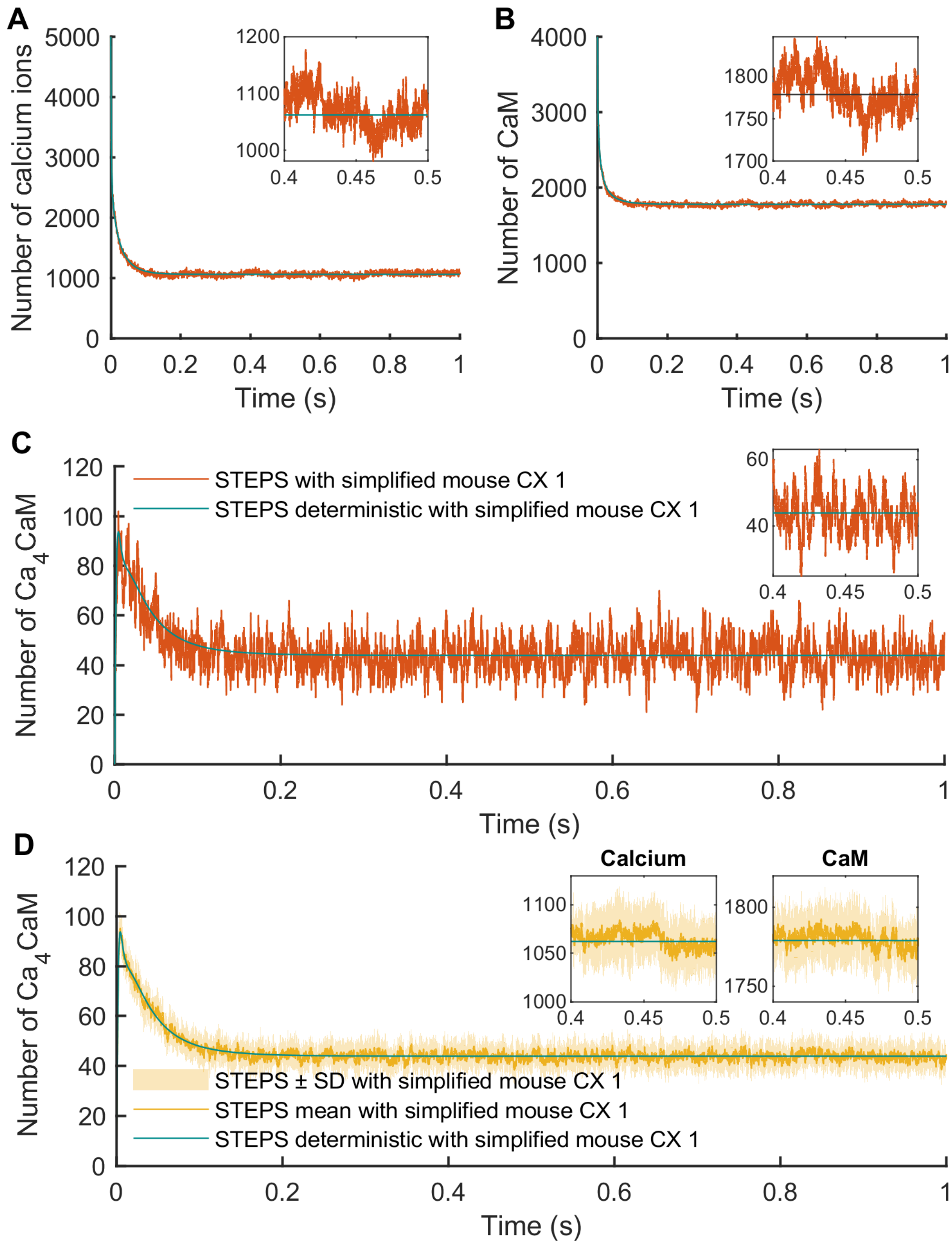

Fig H. Reaction–diffusion simulations with 3D diffusion in the simplified mouse cortical astrocyte branchlet morphology 1 (Simplified mouse CX 1) show consistent results with deterministic and stochastic STEPS. Number of (A) calcium ions, (B) calmodulin (CaM) molecules, and (C) Ca_4CaM complexes plotted as a function of time from a single realization of a stochastic run with STEPS together with deterministic STEPS simulation and (D) mean and standard deviation (shaded area indicates ±SD) of Ca_4CaM from 20 STEPS simulation runs together with deterministic STEPS simulation plotted as a function of time with insets for calcium and calmodulin.

References

1. Stiles JR, Van Helden D, Bartol TM, Salpeter EE, Salpeter MM. Miniature endplate current rise times <100 μs from improved dual recordings can be modeled with passive acetylcholine diffusion from a synaptic vesicle. Proc Natl Acad Sci USA. 1996;93(12):5747-52. doi:10.1073/pnas.93.12.5747.

2. Stiles JR, Bartol TM. Monte Carlo methods for simulating realistic synaptic microphysiology using MCell. In: De Schutter E, editor. Computational Neuroscience: Realistic Modeling for Experimentalists. Boca Raton, FL, USA: CRC Press; 2001. p. 87-127.

3. Kerr RA, Bartol TM, Kaminsky B, Dittrich M, Chang JCJ, Baden SB, et al. Fast Monte Carlo simulation methods for biological reaction-diffusion systems in solution and on surfaces. SIAM J Sci Comput. 2008;30(6):3126-49. doi:10.1137/070692017.

4. Hepburn I, Chen W, Wils S, De Schutter E. STEPS: efficient simulation of stochastic reaction-diffusion models in realistic morphologies. BMC Syst Biol. 2012;6:36. doi:10.1186/1752-0509-6-36.

5. Carnevale NT, Hines ML. The NEURON Book. 1st ed. Cambridge, United Kingdom: Cambridge University Press; 2006. doi:10.1017/CBO9780511541612.

6. McDougal RA, Hines ML, Lytton WW. Reaction-diffusion in the NEURON simulator. Front Neuroinform. 2013;7:28. doi:10.3389/fninf.2013.00028.

7. Nguyen TM, Thomas LA, Rhoades JL, Ricchi I, Yuan XC, Sheridan A, et al. Structured cerebellar connectivity supports resilient pattern separation. Nature. 2023;613(7944):543-9. doi:10.1038/s41586-022-05471-w.

8. Bae JA, Baptiste M, Baptiste MR, Bishop CA, Bodor AL, Brittain D, et al. Functional connectomics spanning multiple areas of mouse visual cortex. Nature. 2025;640(8058):435-47. doi:10.1038/s41586-025-08790-w.

9. Shapson-Coe A, Januszewski M, Berger DR, Pope A, Wu Y, Blakely T, et al. A petavoxel fragment of human cerebral cortex reconstructed at nanoscale resolution. Science. 2024;384(6696):eadk4858. doi:10.1126/science.adk4858.

10. Geuzaine C, Remacle JF. Gmsh: a 3-D finite element mesh generator with built-in pre- and post-processing facilities. Int J Numer Methods Eng. 2009;79(11):1309-31. doi:10.1002/nme.2579.
